# Gene-agnostic approaches to treating inherited retinal degenerations

**DOI:** 10.3389/fcell.2023.1177838

**Published:** 2023-04-13

**Authors:** Lindsey A. Chew, Alessandro Iannaccone

**Affiliations:** ^1^ Duke Center for Retinal Degenerations and Ophthalmic Genetic Diseases, Department of Ophthalmology, Duke Eye Center, Duke University School of Medicine, Durham, NC, United States; ^2^ Department of Cell Biology, Duke University School of Medicine, Durham, NC, United States

**Keywords:** inherited retinal degenerations, retinal dystrophies, gene therapy, gene-agnostic, optogenetics, photoreceptor transplantation, retinal prosthetics, stem cells

## Abstract

Most patients with inherited retinal degenerations (IRDs) have been waiting for treatments that are “just around the corner” for decades, with only a handful of seminal breakthroughs happening in recent years. Highlighting the difficulties in the quest for curative therapeutics, Luxturna required 16 years of development before finally obtaining United States Food and Drug Administration (FDA) approval and its international equivalents. IRDs are both genetically and phenotypically heterogeneous. While this diversity offers many opportunities for gene-by-gene precision medicine-based approaches, it also poses a significant challenge. For this reason, alternative (or parallel) strategies to identify more comprehensive, across-the-board therapeutics for the genetically and phenotypically diverse IRD patient population are very appealing. Even when gene-specific approaches may be available and become approved for use, many patients may have reached a disease stage whereby these approaches may no longer be viable. Thus, alternate visual preservation or restoration therapeutic approaches are needed at these stages. In this review, we underscore several gene-agnostic approaches that are being developed as therapeutics for IRDs. From retinal supplementation to stem cell transplantation, optogenetic therapy and retinal prosthetics, these strategies would bypass at least in part the need for treating every individual gene or mutation or provide an invaluable complement to them. By considering the diverse patient population and treatment strategies suited for different stages and patterns of retinal degeneration, gene agnostic approaches are very well poised to impact favorably outcomes and prognosis for IRD patients.

## 1 Introduction

**FIGURE 1:**
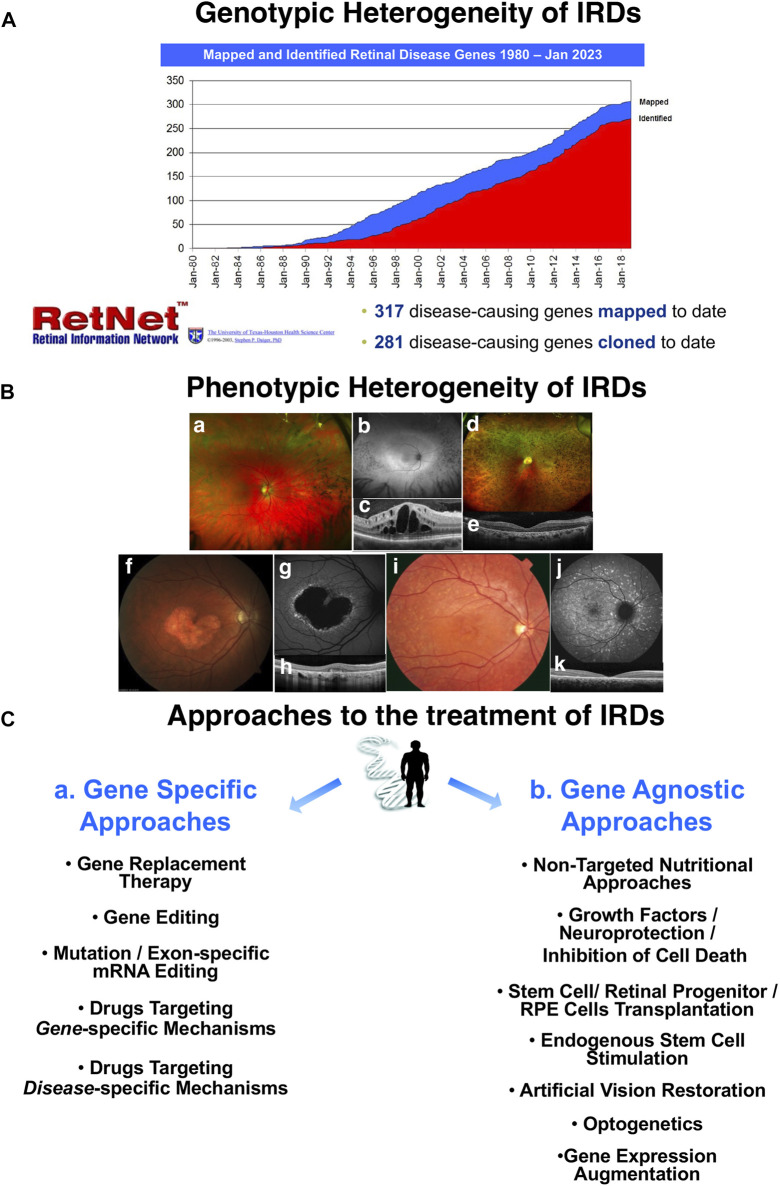
*The landscape of treatment strategies for inherited retinal degenerations (IRD).* Rapid acceleration of research since the 1980s has led to the discovery of 317 disease-causing genes with 281 of these genes now cloned, highlighting genotypic heterogeneity previously described by the RetNet project (hosted by the University of Texas-Houston Health Science Center) **(A)**. The immense phenotypic heterogeneity of IRDs is further illustrated by fundus images **(B, a–k)**. Patients with retinitis pigmentosa (RP) can present with good central retinal preservation **(a–b)** with mild to moderate vision loss and good preservation of the central photoreceptors exemplified by a partially detectable ellipsoid zone (EZ) and retained outer nuclear layer (ONL). However, RP can also induce significant inflammatory complications like cystoid macular edema (CME) **(c)**. In other instances of RP, the macular area may be partially compromised **(d)**, with minimal central EZ preservation **(e)**. In macular, cone, and cone-rod dystrophies, central disruptions are most common, but some present with significant foveal atrophic changes and partial foveal EZ preservation **(f–h)**; others present with global changes marked by central retinal thinning and complete EZ loss **(J–K)**. Gene-specific therapy **(C, a)** is unlikely to benefit patients with severe degeneration **(d–k)**, but gene-agnostic strategies **(C, b)** may still offer these patients a path to recovering vision.

For years, seminal breakthroughs to restore vision have been “just around the corner,” yet most patients with inherited retinal degenerations (IRDs) find themselves continuing to wait. More than 2 decades have passed since the first large animal, Lancelot the Briard dog, was successfully administered gene therapy for Leber’s congenital amaurosis type 2 (LCA2), and his vision was restored (Veske et al., 1999; Lorenz et al., 2000; Bainbridge et al., 2008; Hauswirth et al., 2008; Maguire et al., 2008; Russell et al., 2017). However, this treatment (voretigene neparvovec/Luxturna by Spark Therapeutics in the United States (United States) and Novartis outside the United States) required 16 years before completing extensive trials; Luxturna finally obtained approval from the United States Food and Drug Administration (FDA) and its international equivalents in 2017 (Maguire et al., 2008; Ledford, 2017; Russell et al., 2017). IRDs, are enormously heterogeneous from a genetic standpoint, with over 280 genes cloned to date and over 300 mapped (Figure 1, top). This represents both a great opportunity to deliver gene-by-gene precision medicine-based approaches as it could never be envisioned before, and a major challenge. If genetic heterogeneity was not sufficient, phenotypic heterogeneity adds significantly to the challenge as well (Figure 1B, a-k). Patients with retinitis pigmentosa (RP), the most common form of IRD, can present with good central retinal preservation (Figure 1B, a-b) with mild to moderate vision loss and good preservation of the central photoreceptors exemplified by a partially detectable ellipsoid zone (EZ) and retained outer nuclear layer (ONL) but exhibit important inflammatory complications such as cystoid macular edema (CME, Figures 1B,C), or they can be affected much earlier and more severely in life, whereby the macular area is also partially compromised (Figures 1B,D) and there is minimal central EZ preservation (Figures 1B,E). In macular dystrophies and cone and cone-rod dystrophies, therapeutic help is needed mostly centrally, but a patient could present either with significant atrophic changes around the fovea but partial foveal EZ preservation (Figures 1B,F-H) or could have a more widespread phenotype and, crucially, marked central retinal thinning with complete EZ loss (Figures 1B,J-K). For these patients with exceedingly rare exceptions, gene therapy would be expected to provide no meaningful benefit.

Given the recent advances in our understanding of the mechanisms underlying IRD pathobiology, can we approach IRDs more broadly? Can we use gene-agnostic strategies (Figure 1B) to identify therapeutics that could target a wide range of causative genotypes, therefore bypassing the development of therapies for every individual gene known to impair vision? Where gene-specific therapies already exist, can gene-agnostic strategies still provide complementary support to protect the retina and ultimately preserve vision? While others have tackled similar topics (John et al., 2022), we uniquely explore these questions in the context of IRD progression, examine the intersection of treatment strategies with disease severity, and assess broadly applicable approaches for restoring retinal health—especially those already in or at the cusp of clinical trials.

Several groups have explored this possibility through a wide variety of approaches and ongoing efforts. For example, supplementation with Rod-derived Cone Viability Factor (RdCVF) holds promise for preventing secondary cone demise in primary rod dystrophies (Yang et al., 2009; Aït-Ali et al., 2015; Clérin et al., 2020). Advances in understanding the retina’s neuroimmune interactions and oxidative stress tolerance provide alternative avenues for maintaining retinal health (Wang et al., 2020; Wu et al., 2021), especially in the context of microglial maintenance and antioxidant cocktails (Yu et al., 2004; Shen et al., 2005; Komeima et al., 2006; Komeima et al., 2007; Punzo et al., 2012; O’Koren et al., 2019). Given that the vast number of disease-causing mutations are in genes selectively or primarily expressed by photoreceptors (Rosenfeld et al., 1992; McLaughlin et al., 1993), photoreceptor transplantation has garnered enthusiasm as a therapeutic solution (Comyn et al., 2010; Garita-Hernandez et al., 2021; Chiang and Chern, 2022), especially in combination with induced pluripotent stem cells (iPSCs) (Zhong et al., 2014; Takagi et al., 2019; Watari et al., 2023), embryonic stem cells (ESCs) (Da Cruz et al., 2018), mesenchymal stem cells (Bartsch et al., 2008; Gasparini et al., 2019; Mahato et al., 2020; Sharma and Jaganathan, 2021; Zerti et al., 2021) (MSCs), chemically-induced photoreceptor-like cells (CiPCs) (Mahato et al., 2020) or neural stem cells (Liu et al., 2003; Coles et al., 2004; Frøen et al., 2013). Transplanting retinal pigmented epithelial (RPE) cells also warrants consideration, given the critical role of the RPE in providing the photoreceptors with the necessary support for maintaining the visual cycle and recycling metabolites.

There have been notable achievements in the application of optogenetics *via* adeno-associated viral vectors (AAV) to restore vision to patients, while bypassing photoreceptors entirely (Batabyal et al., 2021a; Gauvain et al., 2021; Sahel et al., 2021). Optogenetic strategies take advantage of opsins, light-sensitive proteins often derived from bacteria, to repurpose them for scientific and medical applications. Depending on the particular opsin, this strategy can require partnership with an active stimulative device (such as goggles) (Gauvain et al., 2021; Sahel et al., 2021). For some patients, this additional equipment may be cumbersome. For this reason, exploration of opsins activated by ambient light is desirable and has been proposed in combination with gene therapy targeting ON bipolar cells (Batabyal et al., 2021b). Fusing optogenetics with photoreceptor transplantation has also generated interest, with some progress made by expressing microbial opsins in neonatal murine photoreceptors, which are then transplanted into a mouse model of retinal degeneration (Garita-Hernandez et al., 2019; 2021). The host immune response following both transplantation and AAV delivery remains a concern. However, an adjacent method employing laser assistance for nano-enhanced optical delivery appears capable of similarly facilitating gene delivery to targeted cell populations without instigating an undesirable immune response (Batabyal et al., 2019; Batabyal et al., 2021b). This reduces the likelihood of adverse events while maximizing the therapeutic potential.

These strategies sharply contrast with artificial vision mediated by retinal and cortical prosthetics (Zhou et al., 2013; Farvardin et al., 2018; Niketeghad and Pouratian, 2019; Pio-Lopez et al., 2021). Requirements for successful application of these prosthetics include surgical implantation and multiple pieces of equipment, such as a specialized camera attached to glasses and a video processing unit. As we approach 10 years since the first implantation of the Argus II retinal prosthetic device, clinical outcomes and patient tolerability of this system vis-à-vis limited benefits have represented a barrier to its continuation alongside cost considerations and the complexities of subsequent training and rehabilitation protocols (Vaidya et al., 2014; Berger et al., 2016; Ghodasra et al., 2016; Ostad-Ahmadi et al., 2021). Thus, simpler systems are required, and continued development of retinal and cortical prosthetics remains ongoing and very important.

As IRD patients continue to experience progressive vision loss, we must adopt a sense of urgency and aspire to a research landscape where the next-generation of therapeutics is five instead of 16 years away. While important, tackling IRDs on an individual, mutation-by-mutation or gene-by-gene basis is unlikely to be the most expedient path forward. With the approval of Luxturna, the field is well-poised to tackle these challenges. By centering our commentary on gene-agnostic approaches to treating IRDs, we focus on more broadly applicable treatment strategies that will expedite and increase treatment access for diverse IRD patients across the globe.

## 2 A photoreceptor survival guide

**FIGURE 2:**
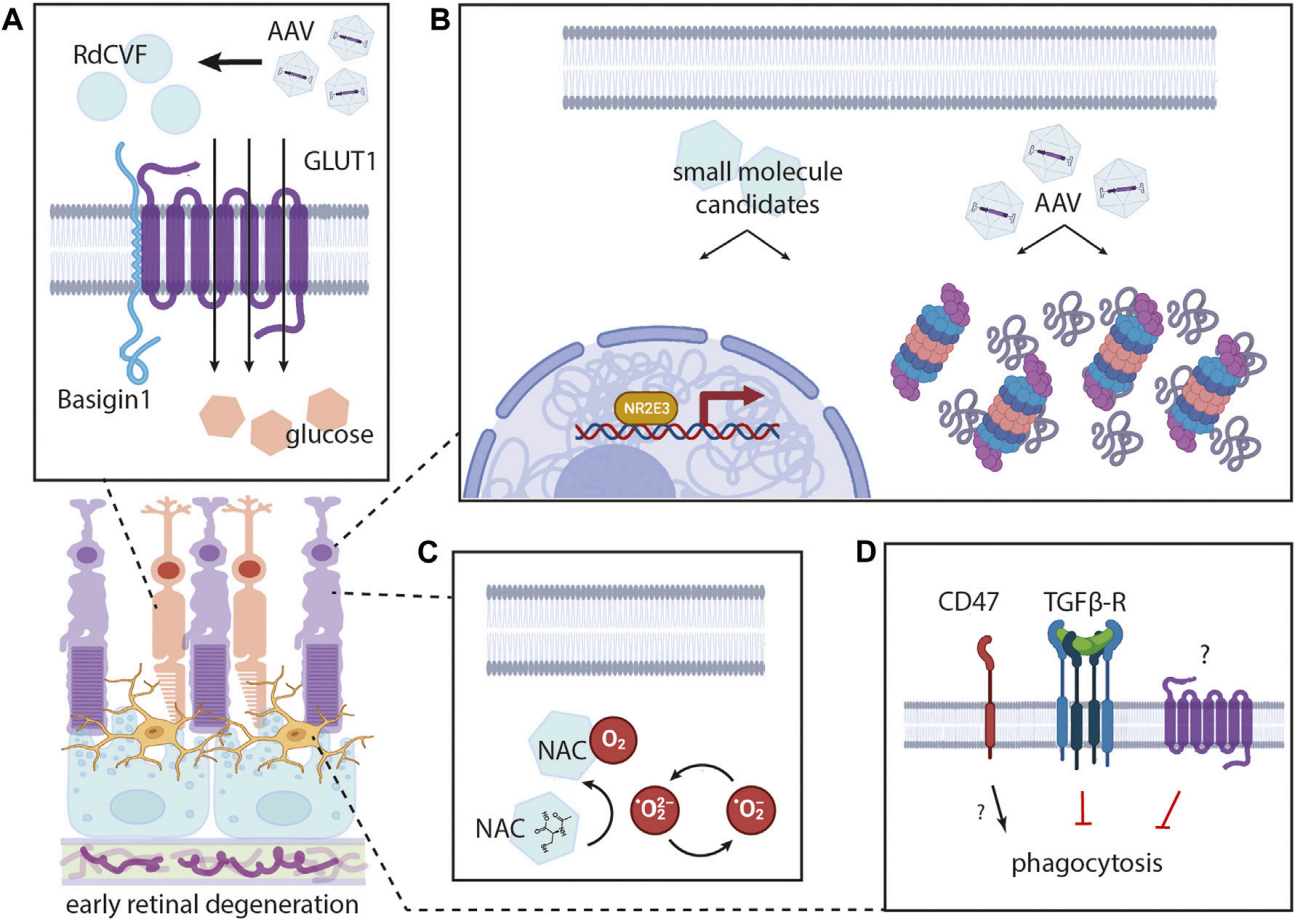
*Mechanisms for enhancing photoreceptor survival in early retinal degeneration.* Introducing Rod-derived Cone Viability Factor (RdCVF) **(A)** through use of AAVs represents one potential strategy for promoting cone photoreceptor survival. The working model for this mechanism includes activation of the Basigin-1/GLUT1 complex to increase transport of glucose and potentially other similar metabolites. Proteasomal enhancement (**(B)** *, right*) to clear excess misfolded proteins represents another possible approach. This strategy could be harnessed through the discovery of small molecules or through gene augmentation for proteasomal machinery. These approaches could also be used for augmenting certain nuclear hormone receptors (like NR2E3) (**(B)** *, left*). Oral supplementation with antioxidants like N-Acetylcysteine (NAC) **(C)** may also support photoreceptor health, with NAC as a known scavenger of reactive oxygen species. Alternative strategies include limiting activation of microglia **(D)**, by inhibiting excessive phagocytosis (*i.e.,* inhibiting the TGFβ receptor), while harnessing their role in maintenance of the retina.

Patients in early and intermediate stages of IRD progression may still retain intact photoreceptors. For these individuals, minimally invasive treatments (Figure 2) that can rescue vision or reduce the risk of further vision loss are especially desirable and feasible. We discuss several promising developments that may support patients’ existing rod and cone photoreceptors to limit further cell death (Aït-Ali et al., 2015; Lobanova et al., 2018; Vighi et al., 2018) and deterioration of visual function. While supporting retinal health may be insufficient to improve patients’ visual acuity, maintaining photoreceptor survival prevents critical and significant declines in visual function. To this end, each of the following approaches aim to reduce the likelihood of photoreceptor cell death. Although we do not provide an exhaustive list, these strategies have reached the clinical trial stage, or will imminently achieve that status, and therefore warrant consideration additional consideration.

### 2.1 Rod-derived cone viability factor (RdCVF)

IRDs involving primary rod photoreceptor death and secondary cone demise have a unique presentation. At times, even patients experiencing late stages of disease with advanced cone loss may continue to retain substantial visual acuity (Cideciyan et al., 1998; Iannaccone et al., 2006). For this reason, supporting the health and function of cone photoreceptors is an extraordinary opportunity for vision preservation in the many such patients worldwide (Wright, 1997).

*In vitro* observations of significantly higher cone photoreceptor survival in the presence of rod photoreceptors, as compared to cultures deprived of rods, have given basis for the idea of a “diffusible trophic factor … released from … rod cells” (Mohand-Said et al., 1998). Rod photoreceptors are approximately 20 times more numerous than cones in many mammalian species (Rodieck, 1998). However, the hypotheses of rod death simply resulting in structural collapse of cones, or causing cone death by toxic byproduct release are contravened by a lack of widespread cone demise immediately following rod death (Hicks and Sahel, 1999). Furthermore, the insoluble glycocalyx surrounding each cone provides significant structural integrity and a link to the nearby RPE cells (Hicks and Sahel, 1999). Eventually the existence of Rod-derived Cone Viability Factor (RdCVF) was discovered (Léveillard et al., 2004).

RdCVF shares 33% similarity with thioredoxin but does not have detectable oxidoreductase activity (Léveillard et al., 2004). Evidence suggests that RdCVF binds to a complex formed by the photoreceptor-specific transmembrane protein Basigin-1 and glucose transporter GLUT1 (Figure 2A) (Aït-Ali et al., 2015). This binding appears to promote cone survival by stimulating intracellular glucose uptake and increasing aerobic glycolysis (Aït-Ali et al., 2015). A redox-sensitive interaction between RdCVF and Basigin-1 might even serve as a prerequisite for full activation of GLUT1 transport activity in photoreceptors (Cepko and Punzo, 2015). Complementary findings indicate that lactose supports photoreceptor health *in vitro* and point to a supportive role of glucose, lactose, and other similar metabolites (Jablonski et al., 2001; Wang et al., 2003). Demonstrating functional RdCVF-mediated cone rescue in a rhodopsin P23H rat model of retinitis pigmentosa served as another critical milestone (Yang et al., 2009; Léveillard et al., 2014). Understanding RdCVF’s mechanism of action has underscored its salient role in retinal physiology in health and disease, ultimately reinforcing the potential for RdCVF’s use in therapies for IRDs (Sahel et al., 2001; Chalmel et al., 2007). At this time, SparingVision is leading multiple preclinical studies to translate these findings into clinical solutions, with advancement to clinical trials in the near future.

### 2.2 Cellular stress management

#### 2.2.1 Oxidative stress, N-Acetylcysteine (NAC), and antioxidant supplementation

There is growing clinical interest across many disciplines in the management of oxidative stress to restore cellular homeostasis (Ferrante et al., 1997; Gilgun-Sherki et al., 2001; Ildefonso et al., 2016; Pinilla et al., 2022). Several clinical trials for retinal supplements (*i.e.,* vitamin A, lutein, zeaxanthin, docosahexaenoic acid) have explored their capacity to slow the progression of retinitis pigmentosa (RP) (Iannaccone et al., 2021a). These initial trials paved the way to continued clinical interest across many disciplines in the management of oxidative stress to restore cellular homeostasis in IRDs (Ildefonso et al., 2016; Pinilla et al., 2022). Identification of the Nrf2 pathway and its role in regulation of oxidative stress responses has generated significant attention (Lee et al., 2003; St-Pierre et al., 2006). Consequently, efforts to enhance Nrf2 signaling have been attempted as a mechanism for inhibiting the cellular oxidative stress response. In *rd1* and *rd10* mouse models, AAV-mediated overexpression of Nrf2 in the RPE demonstrated measurable benefits, preserving RPE morphology and increasing survival, improving photoreceptor health, and boosting visual function as measured by optomotor responses (Wu et al., 2021). Given the high baseline metabolic activity of rod photoreceptors, their death in IRDs may lead to an unfettered hyperoxic microenvironment in the outer nuclear layer, thereby inducing oxidative stress in the surviving photoreceptors (Punzo et al., 2012; Wu et al., 2021). To counteract this imbalance, increasing photoreceptor mitochondrial expression of antioxidant proteins, like glutathione peroxidase and superoxide dismutase 2, resulted in delays in retinal degeneration (Lu et al., 2009; Usui et al., 2009).

A well-established antioxidant agent and reactive oxygen species (ROS) scavenger, N-acetylcysteine (NAC) has been studied for over a decade (Lee et al., 2011; Schimel et al., 2011; Raghu et al., 2021), and there is special interest in its therapeutic use as an oral antioxidant supplement) (Iannaccone et al., 2021b). In both the *rd1* and *rd10* mouse models of retinitis pigmentosa, oral NAC reduced cone cell death and preserved cone function by mitigating oxidative damage (Lee et al., 2011). Evidence also suggests that NAC acts mechanistically by scavenging existing ROS and reversing lipid peroxidation to limit further ROS production (Figure 2C) (Schimel et al., 2011). In a human RPE culture model, the degenerative state is known to correlate with reduced glutathione and glutathione peroxidase levels. NAC treatment increased the expression of both of these enzymes, suggesting its capacity to mitigate the overall redox state of cells and reduce oxidative stress (Schimel et al., 2011; Nuhu et al., 2020). In a mouse model of phototoxic retinal degeneration, oral NAC treatment protected the outer nuclear layer and preserved photoreceptor function on electroretinography (Schimel et al., 2011). Preclinical success has advanced this strategy into clinical trials, where oral NAC has already been reported to improve cone function in retinitis pigmentosa patients in a phase I trial (Campochiaro et al., 2020). While this study primarily focused on validating NAC’s safety profile, patients’ best correct visual acuity significantly improved during a 24-week oral NAC treatment period across all cohorts (Campochiaro et al., 2020). In this regimen, patients received 600, 1,200, or 1800 mg of NAC twice daily for 3 months, followed by a 3 times/day regimen for another 3 months (Campochiaro et al., 2020; Kong et al., 2021). Additional retrospective analysis of this study revealed that the higher NAC dosing regimens reduced the risk of macular sensitivity loss (Kong et al., 2021). In the context of RP, there is an ongoing multicenter, randomized, placebo-controlled trial to determine whether oral NAC treatment can improve cone function (NAC-Attack; NCT05537220).

In parallel efforts toward oxidative stress reduction, other investigations have focused on the potential of multiple other exogenous antioxidants, alpha-tocopherol, ascorbic acid, Mn(III)tetrakis (4-benzoic acid) porphyrin, and alpha-lipoic acid (Komeima et al., 2006). Findings suggested that these compounds could also limit oxidative stress to improve cone photoreceptor survival in models of both slowly- and quickly-progressing retinal degeneration (the Q433ter *RHO* and *rd10* mouse models, respectively) (Komeima et al., 2007; Lu et al., 2009; Usui et al., 2009). While global reduction of reactive oxygen species would likely bear significant side effects, given their important signaling functions (Finkel, 2003), tissue specific regulation of oxidative stress could be adapted for use in IRD treatments (Qi et al., 2007; Koilkonda et al., 2010).

#### 2.2.2 Microglial maintenance

Long-standing dogma dictated that microglia in the retina are always detrimental (Itagaki et al., 1989; Yamasaki et al., 2014; Krasemann et al., 2017), with microglial overactivation causing an excessive oxidative stress response and metabolic dysregulation (Block et al., 2007; Smith et al., 2012; Peng et al., 2014; Zhao et al., 2015; Subhramanyam et al., 2019). In the context of the *rd1* and *rd10* mouse models of retinal degeneration, treatment with the anti-inflammatory cytokine transforming growth factor beta (TGF-β) rescued degenerating cones and protected against loss of visual function (Wang et al., 2020). These results suggested broad benefits of TGF-β on cone survival through a mechanism dependent on microglial activation (Wang et al., 2020). However, recent evidence suggests that microglia also occupy a purposeful physiological niche in the healthy retina (Keren-Shaul et al., 2017; O’Koren et al., 2019). In mice, depleting native microglia from the inner plexiform layer of the retina led to a selective reduction in scotopic and photopic b-wave responses without gross changes in synapse number (O’Koren et al., 2019). Evidence also demonstrated that eliminating retinal microglia failed to augment cone survival by a separate CD47-dependent mechanism, which had been previously hypothesized to be mediated by microglia (Wang et al., 2021). This collection of work highlights the complexity of microglia (Figure 2D) and their various roles in the retinal landscape. Further research to selectively promote their role in maintenance while avoiding excess activation may prove fruitful in the development of treatments.

#### 2.2.3 Proteasomal enhancement

Proteasome overload is another common factor in cellular stress, and evidence points to this being a significant contributor to photoreceptor degeneration in IRDs (Tzekov et al., 2011; Lobanova et al., 2013; 2018). This natural sequela follows the accumulation of misfolded and mistrafficked proteins driven by various disease-causing mutations (Kosmaoglou et al., 2008). In the context of retinitis pigmentosa, the frequently diagnosed rhodopsin P23H mutation causes rhodopsin misfolding and accumulation in the endoplasmic reticulum (Illing et al., 2002; Saliba et al., 2002). Ultimately, these stressors induce the unfolded protein response and photoreceptor death (Lin et al., 2007; Gorbatyuk et al., 2010).

In a Gγ1 knockout mouse model of photoreceptor degeneration (Lobanova et al., 2008; Kolesnikov et al., 2011), the degenerative phenotype features the misfolding of proteins like Gβ_1_, which appears to require Gγ1’s chaperone-like activity for correct folding. Cellular stress in this model is derived from accumulation of Gβ_1_ and other misfolded proteins. Promising evidence from this work demonstrated that preventing this accumulation, by reducing Gβ_1_ expression, led to a complete reversal in the degenerative phenotype of the Gγ1^−/−^ mouse (Lobanova et al., 2013). Furthermore, the severity of photoreceptor retinal degeneration correlated with the misfolded protein levels (Lobanova et al., 2013). Subsequent studies have indicated that increasing photoreceptor proteasomal activity can significantly delay retinal degeneration, with the most substantial benefits conferred by overexpression of the 11S proteasome cap subunit PA28α to enhance ubiquitin-independent protein degradation (Lobanova et al., 2018). In a rhodopsin P23H heterozygous mouse model of retinitis pigmentosa, this strategy quadrupled the number of surviving photoreceptors in the inferior retina of 6 month-old mice (Lobanova et al., 2018). Repeating this approach in a mouse model of Bardet-Biedl Syndrome (Zaghloul and Katsanis, 2009; Liu et al., 2014), a multisystemic disorder affecting photoreceptors alongside other ciliated cells, similarly demonstrated a delay in retinal degeneration (Wang, 2022).

Alternatively, phosphorylation of specific downstream targets in the mammalian target of rapamycin complex 1 (mTORC1) pathway also appears to be a viable strategy for increasing proteasomal activity (Wang et al., 2003). By contrast, reducing the proteolytic capacity of photoreceptors through genetic manipulation in otherwise normal retinas induced retinitis pigmentosa-like pathology (Ando et al., 2014). Altogether, this combination of findings heavily emphasizes the critical role of proteostasis in retinal physiology, with significant degenerative consequences stemming from proteasomal overload. The presence of proteasomal machinery in all photoreceptors also augments their attractiveness as therapeutic targets for IRDs, and evidence strongly supports the pursuit of proteasomal enhancement (Figure 2B) as a gene-agnostic treatment strategy.

### 2.3 Nuclear hormone receptor enhancement

Nuclear hormone receptor enhancement (Figure 2B) is another potential strategy for preserving visual function in IRDs. Mutations in the human nuclear hormone receptor gene *NR2E3* (Haider et al., 2000) with two rather different conditions, a recessive one due to insufficient *NR2E3,* resulting in a disorder of photoreceptor cell fate known as the Enhanced S-Cone Syndrome (ESCS) in which rods are replaced by S-cones that remain preserved for extended periods of time (Roman et al., 2019; Iannaccone et al., 2021a)*,* and in a dominant disorder in which abnormal gain of function mutations cause instead a form of RP without enhanced function or selective preservation of the S-cones (S. Li et al., 2021a). In the *rd7* mouse model, which lacks *Nr2e3* and we have shown that faithfully replicated human ESCS (Roman et al., 2019; Batabyal and Kim, 2021), augmenting expression of the nuclear hormone receptor NR1D1 led to histological and molecular restoration of the *rd7* retina (Cruz et al., 2014). However, due to the pleiotropic effects of this gene on photoreceptor health, *NR2E3* has been studied beyond these two conditions and shown to act also as a genetic modifier that can rescue also other forms of RP (Gire et al., 2007). In pursuit of this strategy for restoring vision, Ocugen has initiated an ongoing clinical trial (NCT05203939) in an effort to test the safety and initial efficacy of *NR2E3-*based gene therapy. This trial aims not only to treat ESCS patients and dominant forms of RP linked to *NR2E3* mutations, but is also trying to harness *NR2E3*’s potential to promote homeostasis in the degenerating retina with other forms of RP (S. Li et al., 2021b). If successful, this therapy could become available for not only patients with ESCS, but also for other forms of RP not linked to *NR2E3* mutations.

## 3 Stem Cell Transplantation and Regeneration

**FIGURE 3:**
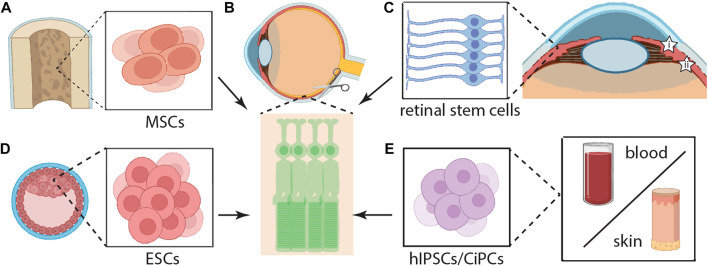
*Stem Cell Transplantation and Regeneration*. Many stem cells sources are being explored, and research efforts are focusing on streamlining regeneration methods to convert these stem cells **(B)** into healthy photoreceptors that can survive subretinal transplantation (*scissors*) while retaining light-responsive properties. Here, we highlight the origin and conversion of the following cells into photoreceptors, mesenchymal stem cells (MSCs) **(A)** *,* retinal stem cells **(C)** from the ciliary margin (*shown by i & ii*), embryonic stem cells (ESCs) **(D)**, human induced pluripotent stem cells (hiPSCs) **(E)**, and chemically-induced photoreceptor-like cells (CiPCs) **(E)** derived from fibroblasts.

As illustrated in Figure 1, patients with end-stage IRDs, who have lost virtually all of their photoreceptors, are outside of the treatment window wherein protective therapies could make a substantive impact. Such patients require restorative approaches that aim to replace rather than protect photoreceptors, or otherwise circumvent their loss. This includes patients with macular dystrophies such as Stargardt disease or related conditions in whom, despite good peripheral retinal functional preservation, central visual loss may be associated with irreparable photoreceptor loss and restorative approaches are needed as well. To this end, numerous stem cell research (Figure 3) efforts have focused on photoreceptor differentiation and transplantation. Harnessing stem cell transplantation technologies has the potential to play a key role in the treatment of patients with advanced IRDs.

### 3.1 Photoreceptors and RPE derived from embryonic stem cells (ESCs) or induced pluripotent stem cells (iPSCs)

Early efforts to direct embryonic stem cells into retinal precursors with the competence for photoreceptor differentiation (Ikeda et al., 2005) provided the basis for subsequent development of transplantable retinal sheets (Zhong et al., 2014; Han et al., 2022). Further optimization of these protocols shortened the minimum time to maturation, with expression of putative photoreceptor markers like OPN1SW (the blue cone pigment) and rhodopsin (Mellough et al., 2012). The development of murine ESC- or iPSC-derived 3D retinal sheets (Figures 3D,E) for transplantation into the *rd1* mouse and other models of retinal degeneration advanced these approaches further (Assawachananont et al., 2014; Jayakody et al., 2015; Ribeiro et al., 2021). Evidence from these studies indicated successful establishment of synaptic connections between host-graft bipolar cells and photoreceptors (Assawachananont et al., 2014; Jayakody et al., 2015; Ribeiro et al., 2021), and provide crucial validation for photoreceptor replacement therapy and its potential to rescue cone-mediated vision. Others have identified the capacity of human iPSCs to autonomously proliferate and spontaneously organize themselves into three-dimensional retinal cups containing properly arranged retinal cell types (Zhong et al., 2014). More recently, efforts have identified three-dimensional retinal organoids as a promising graft source for transplantation therapy (Watari et al., 2023). Evidence also points to preclinical stage success storing associated tissue-sheets for 3, 4 days using a novel preservation method, with functional light responses following retinal transplantation in a rodent model (Watari et al., 2023).

In theory, iPSC-derived RPE bears similar potential for clinical application, particularly when differentiation methods generate and maintain the apical-basolateral polarity characteristic of native RPE structure and function (Miyagishima et al., 2016). Donor-to-donor variability impacts iPSC-derived RPE quality and elevates the requirement for validation of individual graft features prior to consideration for clinical application. Select clinical trials have achieved incremental success for this field, and subretinally transplanted ESC- and iPSC-derived RPE improved visual acuity in some patients (Mandai et al., 2017; Da Cruz et al., 2018; Takagi et al., 2019; Li et al., 2021a), including a cohort with Stargardt macular dystrophy. Intravitreal injection of retinal progenitor cells have also been investigated by jCyte, with a Phase 3 trial that is in the planning phase is required to make determinations beyond safety and efficacy (NCT03073733; NCT04604899; NCT02320812). Multiple clinical trials focusing on stem cell-derived RPE for treating IRDs remain ongoing (ReNeuron Limited, NCT02464436; Southwest Hospital, China, NCT02941991, NCT02749734; Centre d’Etude des Cellules Souches, France, NCT03963154). Further evidence from emerging trials will be required to demonstrate that this strategy can be successful in practice and yield significant improvements for patients’ vision.

### 3.2 Mesenchymal stem cell (MSC) transplantation

Multiple characteristics of mesenchymal stem cells (MSCs) make them suitable for clinical use, including their anti-inflammatory properties derived from extracellular vesicle release (Kou et al., 2022) or their enhancement of autophagy pathways (Liu et al., 2020). The latter was highlighted by a study of rat bone marrow-derived MSC rescue (Figure 3A) of outer nuclear layer thickness in an *in vitro* photoreceptor model (Liu et al., 2020). In a sodium iodate model of retinal degeneration, intravitreal injection of human dental pulp-derived MSCs improved retinal function on electroretinography (Alsaeedi et al., 2019). A proteomic study in a N-methyl-N-nitrosourea injury model of retinal degeneration demonstrated that MSC transplantation conferred a protective effect on photoreceptors, which was attributed to attenuated activation of Pdcd4-mediated programmed cell death pathways (Deng et al., 2021). These rudimentary models provide important insight into the potential of MSC transplantation and establish the basis for subsequent advancement to translational models and clinical trials.

In a non-randomized clinical trial for RP patients, intravitreal injection of autologous bone marrow-derived MSCs improved the best-corrected visual acuity of all participants for several months after the procedure (Tuekprakhon et al., 2021). Unfortunately, the improvements were not sustainable, as their visual acuity reverted to baseline within 12 months of treatment (Tuekprakhon et al., 2021). However, subsequent clinical trials in retinitis pigmentosa patients have reported much success following bone marrow-derived MSC transplantation (Adak et al., 2021). A phase III clinical trial involving suprachoroidal injection of umbilical cord-derived MSC also improved patients’ visual acuity through the 6-month follow-up, without changes in average visual field sensitivity or visual evoked potentials (Kahraman and Oner, 2020; Zhao et al., 2020). Sub-tenon injection of Wharton’s jelly-derived MSCs led to improvements in patients’ visual acuity as well, although this study remains ongoing (Özmert and Arslan, 2020). These advances point to the promise of MSC technologies for vision restoration, although long-term efficacy studies will be required to validate the potential of these treatments.

### 3.3 Chemically-induced photoreceptor-like cells (CiPCs)

Recent seminal work has identified a set of five small molecules capable of chemically inducing transformation of fibroblast into photoreceptor-like cells (CiPCs) (Figure 3E) without first reverting them into pluripotent states or employing transcription factors (Mahato et al., 2020). In the *rd1* mouse model, which is characterized by rapid onset retinal degeneration akin to RP, CiPC transplantation into the subretinal space partially restored the pupillary reflex and visual function, as measured by the light-aversion behavioral paradigm (Mahato et al., 2020). In some tests, this effect was detected under scotopic illumination conditions assessing rod-mediated vision (Mahato et al., 2020). Evidence suggests that CiPC development relies on translocation of AXIN2 to the mitochondria to promote reactive oxygen species production and subsequent activation of NFkB and Ascl1 upregulation (Shin et al., 2016; Mahato et al., 2020). While larger preclinical and clinical studies of CiPC will be required to fully assess the potential of this strategy, more efficient conversion of fibroblasts into photoreceptor-like cells may play a key role in lowering costs and increasing patient access to regenerative medicine.

### 3.4 Neural stem cells of the retina

Reports of true retinal stem cells in the adult human eye raise the possibility of an endogenous source for cellular regeneration (Coles et al., 2004; Frøen et al., 2013). Adult neural stem cells do not require reprogramming, and a retinal stem cell population would fall into this category (Liu et al., 2003). A study of human ocular cell types showed that the human eye contains a small population of approximately 10,000 multipotent, retinal stem cells (Figure 3C) with the potential for proliferation and self-renewal (Coles et al., 2004). Capitalizing on this population in the ciliary margin (Figure 3C, i and ii), the biotechnology company Endogena Therapeutics has focused on a regenerative medicine approach to stimulate proliferation and migration of these retinal stem cells as a therapeutic alternative. While this technology remains proprietary, ongoing clinical trials are evaluating the efficacy of several cocktails of intravitreally delivered small molecules (NCT05392751). Activating endogenous retinal stem cell populations for development into photoreceptors would be exciting; however, any genetic defects would persist in this reactivated stem cell population. These cells might mature into photoreceptors and function normally for some time, but the genetic basis for degeneration would remain. Eventually, degeneration would likely occur at the same rate experienced by the patient prior to any intervention. For early onset IRDs, such as LCA2, this strategy may not be viable; however, slower progressing IRDs, like RP, may be well-suited for treatment by this approach. Further studies will be required to assess the feasibility of repeating treatment to activate retinal stem cells multiple times over a patient’s lifetime. Extended-release formulations could also play a role in long-term activation of patients’ retinal stem cells.

## 4 Optogenetic strategies for restoring vision

Optogenetic therapy provides an unparalleled opportunity for restoring vision to patients who have experienced significant photoreceptor cell death. Through AAV-mediated expression of opsins (*i.e.*, channelrhodopsin, ChrimsonR and Opto-mGluR6) in bipolar cells and retinal ganglion cells, direct stimulation of these secondary and tertiary cell types of the neural retina can bypass photoreceptors while maximizing the activation of typical visual circuits of the brain.

### 4.1 Targeting retinal ganglion cells

**FIGURE 4:**
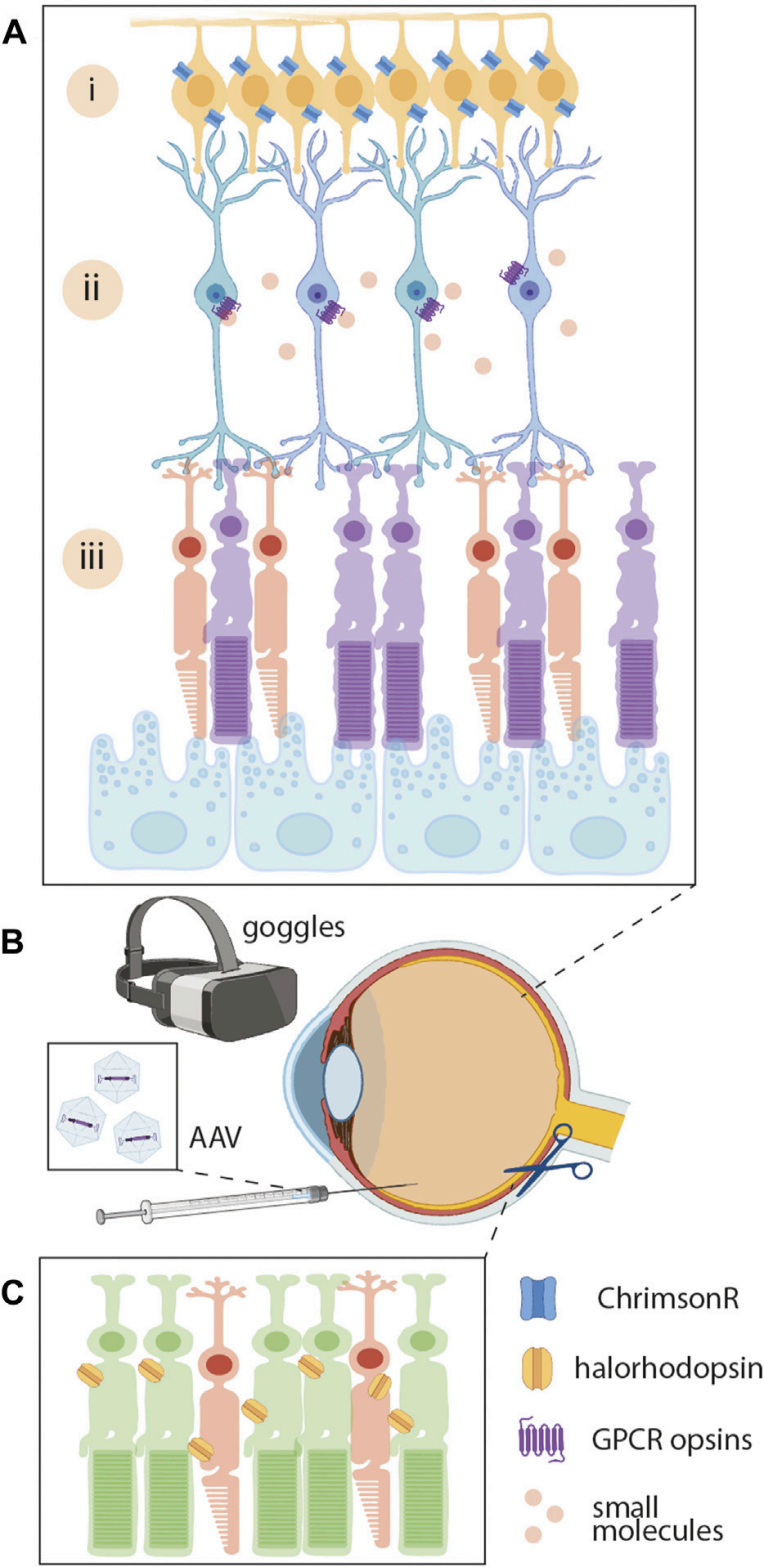
*Optogenetic strategies for restoring vision.* In the degenerating retina **(A)**, dying photoreceptors (iii) fail to detect light or provide the retinal interneurons with signals about visual input. Solutions include targeting retinal ganglion cells (i) with channelrhodopsins (such as ChrimsonR, *blue*). Another option is to target bipolar cells (ii) with GPCR opsins (*purple*) or to combine this approach with small molecules (*red circles*) that further increase light sensitivity. **(B)** Administration of therapy requires intravitreal delivery of AAVs mediating opsin expression in the bipolar cells and retinal ganglion cells. Use of optogenetic goggles convert digital images from a camera into light stimulation of the treated retina to support patients’ restoration of vision **(C)** Another possible solution is to replace dying photoreceptors with a graft of human iPSC-photoreceptors that have been transduced with optogenetic proteins like halorhodopsin.

A series of non-human primate studies established the proof of concept for optogenetic gene therapy targeting retinal ganglion cells (Figures 4A, i) and demonstrated the capacity for AAV-mediated opsin expression without a deleterious immune response (Picaud et al., 2019; Gauvain et al., 2021). Importantly, this work overcame differences in murine and primate immunology, and collaborative efforts identified an AAV-variant (AAV2.7m8) that could effectively transduce retinal ganglion cells following intravitreal injection (Dalkara et al., 2013; Sahel et al., 2021). Translating this strategy into clinic has been enormously successful and restored significant visual perception in a patient with RP, whose visual acuity had been limited to light perception for over a decade (Sahel et al., 2021). By pairing intravitreal injections with active stimulation goggles designed exclusively for converting camera inputs into opsin stimulation, this therapy maximizes the functionality of the newly expressed opsins in patients’ retinal ganglion cells (Gauvain et al., 2021; Sahel et al., 2021; Kralik et al., 2022). Physiotherapy also forms a key component of the therapeutic process, and patients must train themselves to interpret the new format of visual information transmitted by optogenetic goggles (GenSight Biologics; NCT03326336). For patients with severe photoreceptor loss, optogenetic therapy represents hope of regaining some level of vision.

Further advances in optogenetic therapy may be capable of improving the best visual acuity that treatment can offer. For example, rather than achieving vision at the level of object localization within arm’s length (Sahel et al., 2021), future therapies may be able to allow for counting fingers at several feet. These seemingly modest improvements would lead to significant practical possibilities for patients.

### 4.2 Amplifying optogenetic signals in bipolar cells

Following severe photoreceptor degeneration, many retinal interneurons remain physiologically and metabolically stable. Imbuing bipolar cells (Figure 4A, ii) with light sensitivity could maximize the utility of native retinal circuits and restore visual function. Inducing the expression of light-sensitive G-protein coupled receptors, such as vertebrate rhodopsin, in bipolar cells represents another attractive alternative for restoring vision (Gaub et al., 2018). Expanding on this concept, Vedere Bio II is also developing a library of intravitreally injectable small molecules to augment the sensitivity of rhodopsin and increase its signal amplification. Together, AAV-mediated delivery of rhodopsin to bipolar cells followed by intravitreally injectable small molecules that can act as a “molecular switch” has the potential to restore patient’s visual function using ambient light.

Protein engineering to improve the kinetics of opsins used in optogenetic therapy may also facilitate improved outcomes. AAV-mediated delivery of Opto-mGluR6, a chimera of melanopsin with the intracellular domains exchanged for those of mGluR6, represents an early foray into this space (Kralik et al., 2022). This design aimed to optimize activation of the Gα_o_ signaling pathway, the G-protein pathway traditionally activated by mGluR6 at the photoreceptor-ON bipolar cell synapse (Nawy, 1999). Harnessing the natural signal amplification capacity of metabotropic receptors makes GPCR opsins approximately 1000-fold more light sensitive compared to traditional channelrhodopsins (Kralik et al., 2022). Targeting bipolar cells also maximizes the capacity of light signals to naturally propagate to diverse retinal ganglion cell populations and their inherently varied receptive fields (Hulliger et al., 2020). The immense potential of this strategy is highlighted by the encouraging results demonstrating improved visual function and contrast sensitivity in optogenetically stimulated *rd1* mice (Kralik et al., 2022).

### 4.3 Optogenetic photoreceptor transplantation

Combining optogenetic therapy with photoreceptor transplantation may represent a viable strategy for restoring function of the entire retinal circuit, even in patients who have experienced significant photoreceptor loss (Garita-Hernandez et al., 2021; Sakai et al., 2022). Optogenetic transduction of human iPSC-photoreceptors with halorhodopsin eNpHR2.0 has shown success in preclinical studies, following transplantation into *rd1* mice, optogenetic stimulation of eNpHR2.0-expressing photoreceptors led to robust activity in downstream retinal ganglion cells (Garita-Hernandez et al., 2019). Furthermore, immunofluorescence studies from treated *rd1* mice revealed the development of synaptic connections between transplanted photoreceptors and host bipolar cells (Garita-Hernandez et al., 2019). These advances may pave the way toward new treatments for patients with late-stage IRDs.

### 4.4 Evolution of gene therapy

Despite the ocular immune privilege, immune consequences of AAV-mediated therapies remain a consideration in the translational vision research community (Taylor, 2016). Attempts to enhance the transduction of retinal cells by increasing viral loads will likely result in higher toxicity (Khabou et al., 2018), as seen in certain studies involving non-human primates (Vandenberghe et al., 2011; Ramachandran et al., 2017; Hinderer et al., 2018). Selection of the ideal promoter based on its activity and cell-type-specific tropism is a key factor in reducing gene therapy-associated toxicity and optimizing its therapeutic effect. For this reason, promoters with activity restricted to specific cell types (such as human rhodopsin (Allocca et al., 2007; Busskamp et al., 2010) [rods], cone arrestin [cones] (Zhu et al., 2002), and bestrophin-1 (Snodderly et al., 1992; Zhu et al., 2002; Xiong et al., 2019) [RPE] promoters) are typically preferred to globally active potent promoters (such as cytomegalovirus immediate-early (Boshart et al., 1985) [CMV] and chicken beta actin (Hitoshi et al., 1991) [CAG] promoters). Cell-specific promoters eliminate the physiologic stress of indiscriminate expression across cell types that leads to treatment toxicity. These findings highlight the need for more detailed examination of tropism and immune responses associated with viral constructs intended for therapeutic use.

To this end, Vedere Bio (whose assets have now been acquired by Novartis) and Vedere Bio II have initiated recent advances harnessing *in vivo-*directed evolution of new AAV capsids significantly expand the therapeutic potential and applications of AAV gene therapy for IRDs (Dalkara et al., 2013). AAV variants capable of delivering gene cargo to the outer retina following intravitreal injection are especially sought after, with dense tissue of the inner limiting membrane posing a major barrier to successful gene therapy for naturally-occurring AAV serotypes (Snodderly et al., 1992; Fischer et al., 2009). By contrast, *in vivo*-directed evolution of new AAV serotypes appears to have partially overcome these obstacles, and in mouse models of X-linked retinoschisis and LCA2, novel AAV2.7m8 mediated highly efficient gene delivery across retinal layers (Dalkara et al., 2013). Furthermore, AAV2.7m8 facilitated successful transduction of primate photoreceptors following intravitreal injection (Dalkara et al., 2013). Spearheading a segment of these efforts, GenSight Biologics is already invested in ongoing clinical trials (NCT03326336) to integrate this technology with optogenetic approaches.

Continued improvement of AAVs toward increasingly efficient transduction of the outer retina and RPE will be fundamental to the future of gene therapy for IRDs. In combination with selective promoters to achieve minimal toxicity, these novel AAV serotypes will also increase treatment accessibility by enabling patients to receive injections in-office as opposed to in the operating room. Multiple surgical steps that carry significant risks will also be avoided, such as vitrectomy and retinotomy associated with subretinal injections.

### 4.5 Gene therapy without AAV, without goggles, and without immune rejection

For some patients, cytotoxicity may eliminate AAV-mediated gene therapy as an option. A novel method for nano-enhanced optical delivery may alleviate these concerns and serve as a laser-assisted gene therapy alternative (Batabyal et al., 2021a). Known as optoporation, this strategy depends on a pulsed femtosecond near-infrared laser microbeam to facilitate high-efficiency, transient perforations in the cell membrane; this leads to spatially localized transfection of cells with the desired genetic material (Matsuda and Cepko, 2004; Lagali et al., 2008; Doroudchi et al., 2011; Mohanty, 2012). In several optogenetic studies utilizing this method, the neural retina of *rd10* mice remained healthy following optoporation of multicharacteristics opsin (MCO1), a broad-band activatable white-opsin that can be reliably stimulated by ambient light (Batabyal et al., 2015; 2019). In previous work, MCO1-treated mice also showed improvements in visually guided behaviors like the Morris water maze (Wright et al., 2017). This was true even under illumination levels ten times lower than the thresholds typically required for channelrhodopsin stimulation in traditionally optogenetically modified mice (Wright et al., 2017; Batabyal et al., 2021a; Batabyal et al., 2021b). Furthermore, chronic ambient light exposure for 8 h per day did not induce photobleaching in the treated mice (Batabyal et al., 2021b). Nanoscope Therapeutics has launched subsequent clinical trials based on this technology (NCT05417126; NCT04945772). These promising studies may lead to novel therapies that do not require active stimulation goggles, while nano-enhanced optical delivery may obviate the need for AAV and reduce immune response concerns. Immunogenic risks associated with introducing synthetic opsins will remain, but eliminating the introduction of AAV particles will remove the major exacerbating factor.

## 5 Artificial vision and prosthetics

**FIGURE 5:**
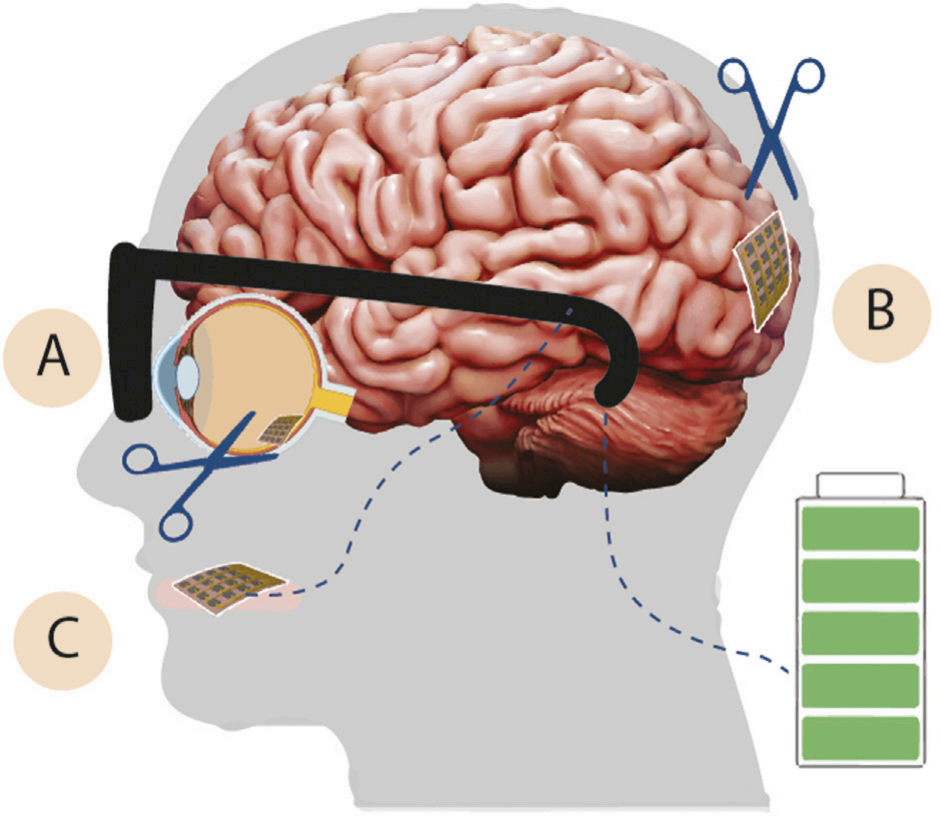
*Artificial visual prosthetic designs.* Goggles with an integrated camera and transmitter (*black*) connected to a battery and visual processing unit (*green, right side*) are required for existing artificial visual prosthetics. The goggles transmit stimulation signals to **(A)** retinal prosthetic devices, surgical implantation (*scissors*) of an electrode array with a receiving coil facilitates stimulation of retinal ganglion cells **(B)** cortical prosthetic devices, surgical implantation of an electrode array in the primary visual cortex (V1) with a receiving coil facilitates stimulation of these cortical neurons **(C)** non-implanted visual prosthetics, placed on the user’s tongue contains integrated receiver for delivering electrical stimulation to the tongue (*pink*).

Developments in artificial vision over the last few decades illustrate significant advances in retinal and cortical prosthetic devices. Creative approaches harness reprogramming of other sensory systems for prosthetics as well. The impressive success of several implantable and wearable prosthetic designs (summarized in Figure 5) warrants particular attention and will be especially valuable for the diverse IRD patient population. In the case of retinal and cortical prosthetics, patients with IRDs often experience a complex conglomeration of symptoms, which makes them candidates for only some of the surgical implantation techniques. The parallel pursuit of multiple prosthetic options will better serve the visually impaired community overall.

### 5.1 Retinal prosthetics

Significant progress in artificial vision research led to approval of Second Sight’s Argus II retinal prosthetic system by the European Union and FDA in 2011 and 2013, respectively (Fernandes et al., 2012; Ghodasra et al., 2016). Argus II, and other similar retinal prosthetics (Figure 5A) required surgical implantation of a receiving coil to the lateral rectus muscle and placement of an electrode array over the macula (Farvardin et al., 2018). As of 2018, four patients had been treated by this strategy and re-gained the ability to perceive hand motions and to perform vague pattern recognition (Farvardin et al., 2018). In addition to the implanted components, these patients used complementary goggles equipped with a sensor-containing camera, a video processing unit, and a transmitting coil (Farvardin et al., 2018). While Argus II featured an array with only 60 electrodes, a next-generation implant in development could incorporate 240 central and peripheral electrodes to improve the resolution of vision (Duncan et al., 2017). Ultimately, electrodes with sizes comparable to that of a retinal ganglion cell would provide optimal vision for the patient (Duncan et al., 2017). However, low resolution of visual restoration has not been the only problem confronting Argus II users. Patients have reported poor battery life for the Argus II system and other features that limit its user friendliness. These are a few reasons why Argus II is no longer available. To address these problems and further enhance this technology, Second Sight (now a division of Vivani Medical) has a new device, Orion II, which is currently in development. These upgrades will be critical to make a retinal prosthetic implant worthwhile for patients, especially given the additional inconveniences posed by surgical implantation, vision rehabilitation, equipment burden, and the associated high costs (Ostad-Ahmadi et al., 2021).

Other prosthetics currently in development include Alpha AMS (Edwards et al., 2018), the Intelligent Retinal Implant System (IRIS V2; NCT02670980), Suprachoroidal Retinal Prosthesis (SRP; NCT01603576), and the PRIMA high-resolution photovoltaic retinal prosthetic system (PRIMA; NCT03333954). The Alpha AMS implant successfully improved visual performance in multiple participants for up to 24 months (Edwards et al., 2018). As with other visual prosthetics, surgical implantation remains challenging, but ongoing improvements in real-time optical coherence tomography microscope guidance and in surgical methods continue to bolster this intervention as a promising option for patients.

The SRP appears to have an acceptable safety profile, yet limitations still exist in the quality of visual contrast discrimination and object localization achieved by this device (Ayton et al., 2014; Slater et al., 2015). Results appear especially mixed in terms of the patients’ ability to use the SRP to identify meaningful characters and localize objects (Shivdasani et al., 2017).

The PRIMA system targets patients with dry age-related macular degeneration (AMD) (Hageman et al., 2005; Toomey, 2015; Landowski et al., 2019), not IRDs; however, advances in this system are likely to become applicable to IRD patients as well. PRIMA has been shown in clinical trials to restore vision in the central scotoma of several patients (Palanker et al., 2020). Importantly, visual acuity correlated with the expectations derived from the pixel pitch (20/420) of the PRIMA device, and higher resolution of pixels in future versions of the implant will likely further improve the maximum achievable visual acuity (Palanker et al., 2020; 2022).

### 5.2 Cortical prosthetics

Cortical visual prosthetics have also been in development for many decades (Dobelle and Mladejovsky, 1974; Dobelle et al., 1976). Recent progress in electrode design, wireless power, and data transmission has generated devices that can support much higher resolution of vision and greater implant stability (Kim et al., 2006; Rush and Troyk, 2012; PR, 2017; Niketeghad and Pouratian, 2019; Pio-Lopez et al., 2021). Importantly, cortical prosthetics implanted in the primary visual cortex (Figure 5B) can also bring vision to patients who are not candidates for retinal implants. This includes patients without functional retinal tissue due to severe IRD progression, as well as those with dysfunctional image forming corticothalamic pathways (*i.e.,* optic nerve-lateral geniculate nucleus-visual cortex) due to developmental and neurological disorders or ocular malformations (*i.e.,* microphthalmia*)*.

### 5.3 Non-implanted visual prosthetics

Patients without any functional vision could also benefit from tactile based visual sensory substitution (Figure 5C) using devices like BrainPort Vision Pro, which remains in clinical trials (Nau et al., 2013; Lee et al., 2014; Stronks et al., 2016; Grant et al., 2018). BrainPort Vision Pro converts image information from a video camera into electrical stimulation patterns emitted from an oral device placed on the user’s tongue (Nau et al., 2013; Lee et al., 2014; Stronks et al., 2016; Grant et al., 2018). Users learn to interpret the electrical stimulation patterns as visual stimuli including shapes, sizes, relative location, and object motion over the course of multiple training sessions, typically totaling 10 hours of one-on-one training over 3 days (Nau et al., 2013; Lee et al., 2014; Stronks et al., 2016; Grant et al., 2018). This strategy does not require any surgical implantation, and the intraoral device can be easily removed for cleaning (Lee et al., 2014; Grant et al., 2018). BrainPort Vision Pro likely harnesses synaptic plasticity in the brain to modulate the visual cortex in a manner conceptually similar to tactile interpretation of Braille lettering (Bola et al., 2017; Silson et al., 2022). BrainPort Vision Pro is currently available in China, with an ongoing clinical trial in the United States (NCT04725760) and expansion efforts in the European Union following Conformité Européene (CE) Mark approval. This technology augments visual prosthetic devices and is currently intended for use alongside traditional assistive technologies like the white cane or a guide dog.

## 6 Discussion

There are many viable, gene-agnostic strategies for treating IRDs. While most of them are best suited for treating patients at a particular stage of disease progression, in combination, they could constitute a powerful arsenal for maintaining or restoring vision across the disease-stage spectrum.

For patients who remain early in their IRD diagnosis, oral supplementation with NAC or other antioxidant cocktails remains a very useful therapeutic option. Mounting evidence in ongoing clinical trials suggests good efficacy for this strategy. Given that NAC and many other supplements can be self-administered by patients daily and at home, adherence to these regimens will likely be high. Delivery of RdCVF or proteasomal enhancers to patients with early disease represents a more definitive opportunity to prevent or at least delay otherwise inevitable visual impairment. Compliance with these treatments is likely to be high for stand-alone delivery by intravitreal injection, while extended-release formulations or AAV-based approaches may limit the burden of frequent repeat treatments.

For IRD patients with intermediate stage of disease progression, combinatorial treatment is even more likely to provide the necessary synergy to restore or protect visual function. In some instances, subretinal surgical implantation of ESCs, iPSCs, or CiPCs may be necessary to augment native photoreceptors. However, surgical and immune rejection risks can be significant, making some patients poor candidates for these procedures. By contrast, intravitreal delivery of a small molecule cocktail is much more accessible for most patients, and activation of a patient’s own retinal stem cells eliminates immune rejection concerns. For patients with early onset disease that obliterates function of their own retinal stem cells, optogenetic strategies targeting bipolar cells or retinal ganglion cells remain gene-agnostic without relying on a patient’s degenerating photoreceptors. Advances in AAV design may also allow the field to transition from subretinal to intravitreal treatment delivery. Optoporation or electro-transfection *via* a proprietary injection system (ETIS) developed by Eyevensys represent competing delivery strategies for both gene therapies and pharmacological/neuroprotective approaches to IRDs. Furthermore, improvements in the design of synthetic opsins may eventually support IRD patients’ ability to regain vision without relying on goggles, which are traditionally required by optogenetic therapies.

For advanced-stage IRD patients with significant outer retina loss, vision restoration will focus on bipolar cells, retinal ganglion cells and the image-forming pathways in the brain. Optogenetic technologies would be ideal for patients whose bipolar cells and retinal ganglion cells remain responsive. By contrast, implantable retinal and cortical visual prosthetics are best suited to create artificial vision in patients whose inner retinal function is compromised as well. Creative approaches using tactile devices, like BrainPort Vision Pro, can also expand the population of patients for whom vision restoration is possible.

From retinal supplementation and stem cell transplantation to optogenetic therapy and retinal prosthetics, a variety of creative strategies hold promise in the quest to protect or restore vision for a broad population of people living with IRDs. Focusing on gene-agnostic approaches to treating IRDs will expedite the development of meaningful therapeutic solutions for patients. Distinct approaches will be suited to IRD patients at various stages of disease progression. Other aspects of health and financial access may also contribute to the “best treatment” for a given patient. Gene specific approaches represent the ultimate example of precision medicine and remain highly desirable and critically important to pursue. However, by investigating common targetable disease pathways and putting sufficient parallel emphasis on the development of gene-agnostic IRD therapeutics as well, we can hope to achieve the long-promised “just around the corner” treatments in time to make a difference for the vast majority of IRD patients.

## References

[B1] Adak S. Magdalene D. Deshmukh S. Das D. Jaganathan B. G. (2021). A review on mesenchymal stem cells for treatment of retinal diseases. Stem Cell Rev. Rep. 17 (4), 1154–1173. 10.1007/S12015-020-10090-X 33410097 PMC7787584

[B2] Aït-Ali N. Fridlich R. Millet-Puel G. Clérin E. Delalande F. Jaillard C. (2015). Rod-derived cone viability factor promotes cone survival by stimulating aerobic glycolysis. Cell 161 (4), 817–832. 10.1016/J.CELL.2015.03.023 25957687

[B3] Allocca M. Mussolino C. Garcia-Hoyos M. Sanges D. Iodice C. Petrillo M. (2007). Novel adeno-associated virus serotypes efficiently transduce murine photoreceptors. J. virology 81 (20), 11372–11380. 10.1128/JVI.01327-07 17699581 PMC2045569

[B4] Alsaeedi H. A. Koh A. E. H. Lam C. Rashid M. B. A. Harun M. H. N. Saleh M. F. B. M. (2019). Dental pulp stem cells therapy overcome photoreceptor cell death and protects the retina in a rat model of sodium iodate-induced retinal degeneration. J. Photochem. Photobiol. B, Biol. 198, 111561. 10.1016/J.JPHOTOBIOL.2019.111561 31352000

[B5] Ando R. Noda K. Tomaru U. Kamoshita M. Ozawa Y. Notomi S. (2014). Decreased proteasomal activity causes photoreceptor degeneration in mice. Investigative Ophthalmol. Vis. Sci. 55 (7), 4682–4690. 10.1167/IOVS.13-13272 PMC4116152 24994871

[B6] Assawachananont J. Mandai M. Okamoto S. Yamada C. Eiraku M. Yonemura S. (2014). Transplantation of embryonic and induced pluripotent stem cell-derived 3D retinal sheets into retinal degenerative mice. Stem Cell Rep. 2 (5), 662–674. 10.1016/J.STEMCR.2014.03.011 PMC4050483 24936453

[B7] Ayton L. N. Blamey P. J. Guymer R. H. Luu C. D. Nayagam D. A. X. Sinclair N. C. (2014). First-in-Human trial of a novel suprachoroidal retinal prosthesis. PLoS ONE 9 (12), 115239. 10.1371/JOURNAL.PONE.0115239 PMC4270734 25521292

[B8] Bainbridge J. W. B. Smith A. J. Barker S. S. Robbie S. Henderson R. Balaggan K. (2008). Effect of gene therapy on visual function in Leber’s congenital amaurosis. N. Engl. J. Med. 358 (21), 2231–2239. 10.1056/NEJMOA0802268 18441371

[B9] Bartsch U. Oriyakhel W. Kenna P. F. Linke S. Richard G. Petrowitz B. (2008). Retinal cells integrate into the outer nuclear layer and differentiate into mature photoreceptors after subretinal transplantation into adult mice. Exp. eye Res. 86 (4), 691–700. 10.1016/J.EXER.2008.01.018 18329018

[B10] Batabyal S. Cervenka G. Ha J. H. Kim Y. T. Mohanty S. (2015). Broad-band activatable white-opsin. PloS one 10 (9), e0136958. 10.1371/JOURNAL.PONE.0136958 26360377 PMC4567350

[B11] Batabyal S. Gajjeraman S. Bhattacharya S. Wright W. Mohanty S. (2019). Nano-enhanced optical gene delivery to retinal degenerated mice. Curr. Gene Ther. 19 (5), 318–329. 10.2174/1566523219666191017114044 31625475 PMC7258984

[B12] Batabyal S. Gajjeraman S. Pradhan S. Bhattacharya S. Wright W. Mohanty S. (2021b). Sensitization of ON-bipolar cells with ambient light activatable multi-characteristic opsin rescues vision in mice. Gene Ther. 28 (3–4), 162–176. 10.1038/S41434-020-00200-2 33087861 PMC9191254

[B13] Batabyal S. Kim S. (2021). Layer-specific nanophotonic delivery of therapeutic opsin-encoding genes into retina. Exp. eye Res. 205, 108444. 10.1016/J.EXER.2021.108444 33516760 PMC9191255

[B14] Batabyal S. Kim S. Wright W. Mohanty S. (2021a). Laser-assisted targeted gene delivery to degenerated retina improves retinal function. J. Biophot. 14 (1), e202000234. 10.1002/JBIO.202000234 33026157

[B15] Berger A. Devenyi R. Héon E. (2016). Retinal prosthesis system for advanced retinitis pigmentosa, A health technology assessment. Ont. Health Technol. Assess. Ser. 16 (14), 1–63. PMC4947979 27468325

[B16] Block M. L. Zecca L. Hong J. S. (2007). Microglia-mediated neurotoxicity, uncovering the molecular mechanisms. Nat. Rev. Neurosci. 8 (1), 57–69. 10.1038/NRN2038 17180163

[B17] Bola Ł. Siuda-Krzywicka K. Paplińska M. Sumera E. Zimmermann M. Jednoróg K. (2017). Structural reorganization of the early visual cortex following Braille training in sighted adults. Sci. Rep. 7 (1), 17448. 10.1038/S41598-017-17738-8 29234091 PMC5727097

[B18] Boshart M. Weber F. Jahn G. Dorsch-Häsler K. Fleckenstein B. Schaffner W. (1985). A very strong enhancer is located upstream of an immediate early gene of human cytomegalovirus. Cell 41 (2), 521–530. 10.1016/S0092-8674(85)80025-8 2985280

[B19] Busskamp V. Duebel J. Balya D. Fradot M. Viney T. J. Siegert S. (2010). Genetic reactivation of cone photoreceptors restores visual responses in retinitis pigmentosa. Science 329 (5990), 413–417. 10.1126/SCIENCE.1190897 20576849

[B20] Campochiaro P. A. Iftikhar M. Hafiz G. Akhlaq A. Tsai G. Wehling D. (2020). Oral N-acetylcysteine improves cone function in retinitis pigmentosa patients in phase I trial. J. Clin. investigation 130 (3), 1527–1541. 10.1172/JCI132990 PMC7269599 31805012

[B21] Cepko C. Punzo C. (2015). Cell metabolism, Sugar for sight. Nature 522 (7557), 428–429. 10.1038/522428a 26108850

[B22] Chalmel F. Léveillard T. Jaillard C. Lardenois A. Berdugo N. Morel E. (2007). Rod-derived Cone Viability Factor-2 is a novel bifunctional-thioredoxin-like protein with therapeutic potential. BMC Mol. Biol. 8, 74. 10.1186/1471-2199-8-74 17764561 PMC2064930

[B23] Chiang M. C. Chern E. (2022). Current development, obstacle and futural direction of induced pluripotent stem cell and mesenchymal stem cell treatment in degenerative retinal disease. Int. J. Mol. Sci. 23 (5), 2529. 10.3390/IJMS23052529 35269671 PMC8910526

[B24] Cideciyan A. V. Hood D. C. Huang Y. Banin E. Li Z. Y. Stone E. M. (1998). Disease sequence from mutant rhodopsin allele to rod and cone photoreceptor degeneration in man. Proc. Natl. Acad. Sci. U. S. A. 95 (12), 7103–7108. 10.1073/PNAS.95.12.7103 9618546 PMC22754

[B25] Clérin E. Marussig M. Sahel J. A. Léveillard T. (2020). Metabolic and redox signaling of the nucleoredoxin-like-1 gene for the treatment of genetic retinal diseases. Int. J. Mol. Sci. 21 (5), 1625. 10.3390/IJMS21051625 32120883 PMC7084304

[B26] Coles B. L. K. Angénieux B. Inoue T. Del Rio-Tsonis K. Spence J. R. McInnes R. R. (2004). Facile isolation and the characterization of human retinal stem cells. Proc. Natl. Acad. Sci. U. S. A. 101 (44), 15772–15777. 10.1073/PNAS.0401596101 15505221 PMC524825

[B27] Comyn O. Lee E. MacLaren R. E. (2010). Induced pluripotent stem cell therapies for retinal disease. Curr. Opin. neurology 23 (1), 4–9. 10.1097/WCO.0B013E3283352F96 PMC2896975 19949329

[B28] Cruz N. M. Yuan Y. Leehy B. D. Baid R. Kompella U. DeAngelis M. M. (2014). Modifier genes as therapeutics, the nuclear hormone receptor Rev Erb alpha (Nr1d1) rescues Nr2e3 associated retinal disease. PloS one 9 (1), e87942. 10.1371/JOURNAL.PONE.0087942 24498227 PMC3909326

[B29] Da Cruz L. Fynes K. Georgiadis O. Kerby J. Luo Y. H. Ahmado A. (2018). Phase 1 clinical study of an embryonic stem cell-derived retinal pigment epithelium patch in age-related macular degeneration. Nat. Biotechnol. 36 (4), 328–337. 10.1038/NBT.4114 29553577

[B30] Dalkara D. Byrne L. C. Klimczak R. R. Visel M. Yin L. Merigan W. H. (2013). *In vivo*-directed evolution of a new adeno-associated virus for therapeutic outer retinal gene delivery from the vitreous. Sci. Transl. Med. 5 (189), 189ra76. 10.1126/SCITRANSLMED.3005708 23761039

[B31] Deng C. L. Hu C. B. Ling S. T. Zhao N. Bao L. H. Zhou F. (2021). Photoreceptor protection by mesenchymal stem cell transplantation identifies exosomal MiR-21 as a therapeutic for retinal degeneration. Cell death Differ. 28 (3), 1041–1061. 10.1038/S41418-020-00636-4 33082517 PMC7937676

[B32] Dobelle W. H. Mladejovsky M. G. Evans J. R. Roberts T. S. Girvin J. P. (1976). Braille” reading by a blind volunteer by visual cortex stimulation. Nature 259 (5539), 111–112. 10.1038/259111A0 1246346

[B33] Dobelle W. H. Mladejovsky M. G. (1974). Phosphenes produced by electrical stimulation of human occipital cortex, and their application to the development of a prosthesis for the blind. J. physiology 243 (2), 553–576. 10.1113/JPHYSIOL.1974.SP010766 PMC1330721 4449074

[B34] Doroudchi M. M. Greenberg K. P. Liu J. Silka K. A. Boyden E. S. Lockridge J. A. (2011). Virally delivered channelrhodopsin-2 safely and effectively restores visual function in multiple mouse models of blindness. Mol. Ther. , J. Am. Soc. Gene Ther. 19 (7), 1220–1229. 10.1038/MT.2011.69 PMC3129568 21505421

[B35] Duncan J. L. Richards T. P. Arditi A. da Cruz L. Dagnelie G. Dorn J. D. (2017). Improvements in vision-related quality of life in blind patients implanted with the Argus II Epiretinal Prosthesis. Clin. Exp. optometry 100 (2), 144–150. 10.1111/CXO.12444 PMC5347867 27558213

[B36] Edwards T. L. Cottriall C. L. Xue K. Simunovic M. P. Ramsden J. D. Zrenner E. (2018). Assessment of the electronic retinal implant alpha AMS in restoring vision to blind patients with end-stage retinitis pigmentosa. Ophthalmology 125 (3), 432–443. 10.1016/J.OPHTHA.2017.09.019 29110946 PMC5818267

[B37] Farvardin M. Afarid M. Attarzadeh A. Johari M. K. Mehryar M. Nowroozzadeh M. H. (2018). The Argus-II retinal prosthesis implantation; from the global to local successful experience. Front. Neurosci. 12, 584. 10.3389/fnins.2018.00584 30237759 PMC6136639

[B38] Fernandes R. A. B. Diniz B. Ribeiro R. Humayun M. (2012). Artificial vision through neuronal stimulation. Neurosci. Lett. 519 (2), 122–128. 10.1016/J.NEULET.2012.01.063 22342306

[B39] Ferrante R. J. Browne S. E. Shinobu L. A. Bowling A. C. Baik M. J. MacGarvey U. (1997). Evidence of increased oxidative damage in both sporadic and familial amyotrophic lateral sclerosis. J. Neurochem. 69 (5), 2064–2074. 10.1046/J.1471-4159.1997.69052064.X 9349552

[B40] Finkel T. (2003). Oxidant signals and oxidative stress. Curr. Opin. Cell Biol. 15 (2), 247–254. 10.1016/S0955-0674(03)00002-4 12648682

[B41] Fischer M. D. Huber G. Beck S. C. Tanimoto N. Muehlfriedel R. Fahl E. (2009). Noninvasive, *in vivo* assessment of mouse retinal structure using optical coherence tomography. PloS one 4 (10), e7507. 10.1371/JOURNAL.PONE.0007507 19838301 PMC2759518

[B42] Frøen R. Erik O. J. Bjørn N. Andrea F. Goran P. Morten C. M. (2013). Does the adult human ciliary body epithelium contain “true” retinal stem cells? BioMed Res. Int. 2013, 531579. 10.1155/2013/531579 24286080 PMC3826557

[B43] Garita-Hernandez M. Chaffiol A. Guibbal L. Routet F. Khabou H. Riancho L. (2021). Control of microbial opsin expression in stem cell derived cones for improved outcomes in cell therapy. Front. Cell. Neurosci. 15, 70. 10.3389/fncel.2021.648210 PMC8012682 33815066

[B44] Garita-Hernandez M. Lampič M. Chaffiol A. Guibbal L. Routet F. Santos-Ferreira T. (2019). Restoration of visual function by transplantation of optogenetically engineered photoreceptors. Nat. Commun. 10 (1), 4524–4613. 10.1038/s41467-019-12330-2 31586094 PMC6778196

[B45] Gasparini S. J. Llonch S. Borsch O. Ader M. (2019). Transplantation of photoreceptors into the degenerative retina, Current state and future perspectives. Prog. Retin. eye Res. 69, 1–37. 10.1016/J.PRETEYERES.2018.11.001 30445193

[B46] Gaub B. M. Berry M. H. Visel M. Holt A. Isacoff E. Y. Flannery J. G. (2018). Optogenetic retinal gene therapy with the light gated GPCR vertebrate rhodopsin. Methods Mol. Biol. 1715, 177–189. 10.1007/978-1-4939-7522-8_12 29188513 PMC7307607

[B47] Gauvain G. Akolkar H. Chaffiol A. Arcizet F. Khoei M. A. Desrosiers M. (2021). Optogenetic therapy, high spatiotemporal resolution and pattern discrimination compatible with vision restoration in non-human primates. Commun. Biol. 4 (1), 125. 10.1038/s42003-020-01594-w 33504896 PMC7840970

[B48] Ghodasra D. H. Chen A. Arevalo J. F. Birch D. G. Branham K. Coley B. (2016). Worldwide Argus II implantation, Recommendations to optimize patient outcomes. BMC Ophthalmol. 16 (1), 52–58. 10.1186/s12886-016-0225-1 27154461 PMC4858839

[B49] Gilgun-Sherki Y. Melamed E. Offen D. (2001). Oxidative stress induced-neurodegenerative diseases, the need for antioxidants that penetrate the blood brain barrier. Neuropharmacology 40 (8), 959–975. 10.1016/S0028-3908(01)00019-3 11406187

[B50] Gire A. Sullivan L. S. Bowne S. J. Birch D. G. Hughbanks-Wheaton D. Heckenlively J. R. (2007). The Gly56Arg mutation in NR2E3 accounts for 1-2% of autosomal dominant retinitis pigmentosa. Mol. Vis. 13, 1970–1975. 17982421

[B51] Gorbatyuk M. S. Knox T. LaVail M. M. Gorbatyuk O. S. Noorwez S. M. Hauswirth W. W. (2010). Restoration of visual function in P23H rhodopsin transgenic rats by gene delivery of BiP/Grp78. Proc. Natl. Acad. Sci. U. S. A. 107 (13), 5961–5966. 10.1073/PNAS.0911991107 20231467 PMC2851865

[B52] Grant P. Maeng M. Arango T. Hogle R. Szlyk J. Seiple W. (2018). Performance of real-world functional tasks using an updated oral electronic vision device in persons blinded by trauma. Optometry Vis. Sci. , official Publ. Am. Acad. Optometry 95 (9), 766–773. 10.1097/OPX.0000000000001273 30169354

[B53] Hageman G. S. Anderson D. H. Johnson L. V. Hancox L. S. Taiber A. J. Hardisty L. I. (2005). A common haplotype in the complement regulatory gene factor H (HF1/CFH) predisposes individuals to age-related macular degeneration. Proc. Natl. Acad. Sci. U. S. A. 102 (20), 7227–7232. 10.1073/PNAS.0501536102 15870199 PMC1088171

[B54] Haider N. B. Jacobson S. G. Cideciyan A. V. Swiderski R. Streb L. M. Searby C. (2000). Mutation of a nuclear receptor gene, NR2E3, causes enhanced S cone syndrome, a disorder of retinal cell fate. Nat. Genet. 24 (2), 127–131. 10.1038/72777 10655056

[B55] Han I. C. Bohrer L. R. Gibson-Corley K. N. Wiley L. A. Shrestha A. Harman B. E. (2022). Biocompatibility of human induced pluripotent stem cell-derived retinal progenitor cell grafts in immunocompromised rats. Cell Transplant. 31, 9636897221104451. 10.1177/09636897221104451 35758274 PMC9247396

[B56] Hauswirth W. W. Aleman T. S. Kaushal S. Cideciyan A. V. Schwartz S. B. Wang L. (2008). Treatment of leber congenital amaurosis due to RPE65 mutations by ocular subretinal injection of adeno-associated virus gene vector, short-term results of a phase I trial. Hum. gene Ther. 19 (10), 979–990. 10.1089/HUM.2008.107 18774912 PMC2940541

[B57] Hicks D. Sahel J. A. (1999). The implications of rod-dependent cone survival for basic and clinical research. Investigative Ophthalmol. Vis. Sci. 40, 3071–3074. 10586925

[B58] Hinderer C. Katz N. Buza E. L. Dyer C. Goode T. Bell P. (2018). Severe toxicity in nonhuman primates and piglets following high-dose intravenous administration of an adeno-associated virus vector expressing human SMN. Hum. gene Ther. 29 (3), 285–298. 10.1089/HUM.2018.015 29378426 PMC5865262

[B59] Hitoshi N. Ken-ichi Y. Jun-ichi M. (1991). Efficient selection for high-expression transfectants with a novel eukaryotic vector. Gene 108 (2), 193–199. 10.1016/0378-1119(91)90434-D 1660837

[B60] Hulliger E. C. Hostettler S. M. Kleinlogel S. (2020). Empowering retinal gene therapy with a specific promoter for human rod and cone ON-bipolar cells. Mol. Ther. Methods & Clin. Dev. 17, 505–519. 10.1016/J.OMTM.2020.03.003 32258214 PMC7114634

[B61] Iannaccone A. Alekseev O. Krauss E. (2021b). Retinitis pigmentosa, rare diseases. National Organization of Rare Diseases.

[B62] Iannaccone A. Brabbit E. Lopez-Miro C. Love Z. Griffiths V. Kedrov M. (2021a). Interspecies correlations between human and mouse nr2e3-associated recessive disease. J. Clin. Med. 10 (3), 475–527. 10.3390/JCM10030475 33513943 PMC7865474

[B63] Iannaccone A. Man D. Waseem N. Jennings B. J. Ganapathiraju M. Gallaher K. (2006). Retinitis pigmentosa associated with rhodopsin mutations, Correlation between phenotypic variability and molecular effects. Vis. Res. 46 (27), 4556–4567. 10.1016/J.VISRES.2006.08.018 17014888

[B64] Ikeda H. Osakada F. Watanabe K. Mizuseki K. Haraguchi T. Miyoshi H. (2005). Generation of Rx+/Pax6+ neural retinal precursors from embryonic stem cells. Proc. Natl. Acad. Sci. U. S. A. 102 (32), 11331–11336. 10.1073/PNAS.0500010102 16076961 PMC1183536

[B65] Ildefonso C. J. Jaime H. Brown E. E. Iwata R. L. Ahmed C. M. Massengill M. T. (2016). Targeting the Nrf2 signaling pathway in the retina with a gene-delivered secretable and cell-penetrating peptide. Investigative Ophthalmol. Vis. Sci. 57 (2), 372–386. 10.1167/IOVS.15-17703 PMC5110262 26842755

[B66] Illing M. E. Rajan R. S. Bence N. F. Kopito R. R. (2002). A rhodopsin mutant linked to autosomal dominant retinitis pigmentosa is prone to aggregate and interacts with the ubiquitin proteasome system. J. Biol. Chem. 277 (37), 34150–34160. 10.1074/JBC.M204955200 12091393

[B67] Itagaki S. McGeer P. L. Akiyama H. Zhu S. Selkoe D. (1989). Relationship of microglia and astrocytes to amyloid deposits of Alzheimer disease. J. Neuroimmunol. 24 (3), 173–182. 10.1016/0165-5728(89)90115-X 2808689

[B68] Jablonski M. M. Tombran-Tink J. Mrazek D. A. Iannaccone A. (2001). Pigment epithelium-derived factor supports normal Müller cell development and glutamine synthetase expression after removal of the retinal pigment epithelium. GLIA 35 (1), 14–25. 10.1002/glia.1066 11424188

[B69] Jayakody S. A. Gonzalez-Cordero A. Ali R. R. Pearson R. A. (2015). Cellular strategies for retinal repair by photoreceptor replacement. Prog. Retin. eye Res. 46, 31–66. 10.1016/J.PRETEYERES.2015.01.003 25660226

[B70] John M. C. Quinn J. Hu M. L. Cehajic-Kapetanovic J. Xue K. (2022). Gene-agnostic therapeutic approaches for inherited retinal degenerations. Front. Mol. Neurosci. 15, 1068185. 10.3389/FNMOL.2022.1068185 36710928 PMC9881597

[B71] Kahraman N. S. Oner A. (2020). Umbilical cord derived mesenchymal stem cell implantation in retinitis pigmentosa, a 6-month follow-up results of a phase 3 trial. Int. J. Ophthalmol. 13 (9), 1423–1429. 10.18240/IJO.2020.09.14 32953582 PMC7459232

[B72] Keren-Shaul H. Spinrad A. Weiner A. Matcovitch-Natan O. Dvir-Szternfeld R. Ulland T. K. (2017). A unique microglia type associated with restricting development of alzheimer’s disease. Cell 169 (7), 1276–1290. 10.1016/J.CELL.2017.05.018 28602351

[B73] Khabou H. Cordeau C. Pacot L. Fisson S. Dalkara D. (2018). Dosage thresholds and influence of transgene cassette in adeno-associated virus-related toxicity. Hum. gene Ther. 29 (11), 1235–1241. 10.1089/HUM.2018.144 30132368

[B74] Kim T. Troyk P. R. Bak M. (2006). “Active floating micro electrode arrays (AFMA),” in Conference proceedings, Annual International Conference of the IEEE Engineering in Medicine and Biology Society, United Kingdom, 10 April 2006, 2807–2810. 10.1109/IEMBS.2006.259981 17946982

[B75] Koilkonda R. D. Chou T. H. Porciatti V. Hauswirth W. W. Guy J. (2010). Induction of rapid and highly efficient expression of the human ND4 complex I subunit in the mouse visual system by self-complementary adeno-associated virus. Archives Ophthalmol. Chic. Ill 128 (7), 876–883. 10.1001/ARCHOPHTHALMOL.2010.135 PMC3431796 20625049

[B76] Kolesnikov A. V. Rikimaru L. Hennig A. K. Lukasiewicz P. D. Fliesler S. J. Govardovskii V. I. (2011). G-protein betagamma-complex is crucial for efficient signal amplification in vision. J. Neurosci. , official J. Soc. Neurosci. 31 (22), 8067–8077. 10.1523/JNEUROSCI.0174-11.2011 PMC3118088 21632928

[B77] Komeima K. Rogers B. S. Campochiaro P. A. (2007). Antioxidants slow photoreceptor cell death in mouse models of retinitis pigmentosa. J. Cell. physiology 213 (3), 809–815. 10.1002/JCP.21152 17520694

[B78] Komeima K. Rogers B. S. Lu L. Campochiaro P. A. (2006). Antioxidants reduce cone cell death in a model of retinitis pigmentosa. Proc. Natl. Acad. Sci. U. S. A. 103 (30), 11300–11305. 10.1073/PNAS.0604056103 16849425 PMC1544081

[B79] Kong X. Gulnar H. Dagmar W. Anam A. Peter A. C. (2021). Locus-level changes in macular sensitivity in patients with retinitis pigmentosa treated with oral N-acetylcysteine. Am. J. Ophthalmol. 221, 105–114. 10.1016/j.ajo.2020.08.002 32795434 PMC7736203

[B80] Kosmaoglou M. Schwarz N. Bett J. S. Cheetham M. E. (2008). Molecular chaperones and photoreceptor function. Prog. Retin. eye Res. 27 (4), 434–449. 10.1016/J.PRETEYERES.2008.03.001 18490186 PMC2568879

[B81] Kou M. Huang L. Yang J. Chiang Z. Chen S. Liu J. (2022). Mesenchymal stem cell-derived extracellular vesicles for immunomodulation and regeneration, a next generation therapeutic tool? Cell Death Dis. 13 (7), 580–616. 10.1038/s41419-022-05034-x 35787632 PMC9252569

[B82] Kralik J. van Wyk M. Stocker N. Kleinlogel S. (2022). Bipolar cell targeted optogenetic gene therapy restores parallel retinal signaling and high-level vision in the degenerated retina. Commun. Biol. 5, 1116–1215. 10.1038/s42003-022-04016-1 36266533 PMC9585040

[B83] Krasemann S. Madore C. Cialic R. Baufeld C. Calcagno N. El Fatimy R. (2017). The TREM2-APOE pathway drives the transcriptional phenotype of dysfunctional microglia in neurodegenerative diseases. Immunity 47 (3), 566–581. 10.1016/J.IMMUNI.2017.08.008 28930663 PMC5719893

[B84] Lagali P. S. Balya D. Awatramani G. B. Münch T. A. Kim D. S. Busskamp V. (2008). Light-activated channels targeted to ON bipolar cells restore visual function in retinal degeneration. Nat. Neurosci. 11 (6), 667–675. 10.1038/NN.2117 18432197

[B85] Landowski M. Kelly U. Klingeborn M. Groelle M. Ding J. D. Grigsby D. (2019). Human complement factor H Y402H polymorphism causes an age-related macular degeneration phenotype and lipoprotein dysregulation in mice. Proc. Natl. Acad. Sci. U. S. A. 116 (9), 3703–3711. 10.1073/PNAS.1814014116 30808757 PMC6397537

[B86] Ledford H. (2017). FDA advisers back gene therapy for rare form of blindness. Nature 550 (7676), 314. 10.1038/NATURE.2017.22819 29052639

[B87] Lee J. M. Calkins M. J. Chan K. Kan Y. W. Johnson J. A. (2003). Identification of the NF-E2-related factor-2-dependent genes conferring protection against oxidative stress in primary cortical astrocytes using oligonucleotide microarray analysis. J. Biol. Chem. 278 (14), 12029–12038. 10.1074/JBC.M211558200 12556532

[B88] Lee S. Y. Usui S. Zafar A. B. Oveson B. C. Jo Y. J. Lu L. (2011). N-Acetylcysteine promotes long-term survival of cones in a model of retinitis pigmentosa. J. Cell. physiology 226 (7), 1843–1849. 10.1002/JCP.22508 21506115

[B89] Lee V. K. Nau A. C. Laymon C. Chan K. C. Rosario B. L. Fisher C. (2014). Successful tactile based visual sensory substitution use functions independently of visual pathway integrity. Front. Hum. Neurosci. 8, 291. 10.3389/FNHUM.2014.00291 24860473 PMC4026734

[B90] Léveillard T. Fridlich R. Clérin E. Aït-Ali N. Millet-Puel G. Jaillard C. (2014). Therapeutic strategy for handling inherited retinal degenerations in a gene-independent manner using rod-derived cone viability factors. Comptes rendus Biol. 337 (3), 207–213. 10.1016/J.CRVI.2013.12.002 24702847

[B91] Léveillard T. Mohand-Saïd S. Lorentz O. Hicks D. Fintz A. C. Clérin E. (2004). Identification and characterization of rod-derived cone viability factor. Nat. Genet. 36 (7), 755–759. 10.1038/NG1386 15220920

[B92] Li S. Datta S. Brabbit E. Love Z. Woytowicz V. Flattery K. (2021a). Nr2e3 is a genetic modifier that rescues retinal degeneration and promotes homeostasis in multiple models of retinitis pigmentosa. Gene Ther. 28 (5), 223–241. 10.1038/S41434-020-0134-Z 32123325 PMC7483267

[B93] Li S. Liu Y. Wang L. Wang F. Zhao T. T. Li Q. Y. (2021b). A phase I clinical trial of human embryonic stem cell-derived retinal pigment epithelial cells for early-stage Stargardt macular degeneration, 5-years’ follow-up. Cell Prolif. 54 (9), e13100. 10.1111/CPR.13100 34347352 PMC8450131

[B94] Lin J. H. Li H. Yasumura D. Cohen H. R. Zhang C. Panning B. (2007). IRE1 signaling affects cell fate during the unfolded protein response. Science 318 (5852), 944–949. 10.1126/SCIENCE.1146361 17991856 PMC3670588

[B95] Liu C. Y. Westerlund U. Svensson M. Moe M. C. Varghese M. Berg-Johnsen J. (2003). Artificial niches for human adult neural stem cells, possibility for autologous transplantation therapy. J. hematotherapy stem Cell Res. 12 (6), 689–699. 10.1089/15258160360732713 14977478

[B96] Liu X. Xie J. Yang L. Li Y. He Y. Liu Z. (2020). Bone marrow mesenchymal stem cells enhance autophagy and help protect cells under hypoxic and retinal detachment conditions. J. Cell. Mol. Med. 24 (6), 3346–3358. 10.1111/JCMM.15008 32003125 PMC7131940

[B97] Liu Y. P. Tsai I. C. Morleo M. Oh E. C. Leitch C. C. Massa F. (2014). Ciliopathy proteins regulate paracrine signaling by modulating proteasomal degradation of mediators. J. Clin. investigation 124 (5), 2059–2070. 10.1172/JCI71898 PMC4001542 24691443

[B98] Lobanova E. S. Finkelstein S. Herrmann R. Chen Y. M. Kessler C. Michaud N. A. (2008). Transducin gamma-subunit sets expression levels of alpha- and beta-subunits and is crucial for rod viability. J. Neurosci. , official J. Soc. Neurosci. 28 (13), 3510–3520. 10.1523/JNEUROSCI.0338-08.2008 PMC2795350 18367617

[B99] Lobanova E. S. Finkelstein S. Li J. Travis A. M. Hao Y. Klingeborn M. (2018). Increased proteasomal activity supports photoreceptor survival in inherited retinal degeneration. Nat. Commun. 9 (1), 1738. 10.1038/S41467-018-04117-8 29712894 PMC5928105

[B100] Lobanova E. S. Stella F. Nikolai P. S. Vadim Y. A. (2013). Proteasome overload is a common stress factor in multiple forms of inherited retinal degeneration. Proc. Natl. Acad. Sci. U. S. A. 110 (24), 9986–9991. 10.1073/PNAS.1305521110 23716657 PMC3683722

[B101] Lorenz B. Gyürüs P. Preising M. Bremser D. Gu S. Andrassi M. (2000). Early-onset severe rod–cone dystrophy in young children with RPE65 mutations. Investigative Ophthalmol. Vis. Sci. 41, 2735–2742. 10937591

[B102] Lu L. Oveson B. C. Jo Y. J. Lauer T. W. Usui S. Komeima K. (2009). Increased expression of glutathione peroxidase 4 strongly protects retina from oxidative damage. Antioxidants redox Signal. 11 (4), 715–724. 10.1089/ARS.2008.2171 PMC2787833 18823256

[B103] Maguire A. M. Simonelli F. Pierce E. A. Pugh E. N. Jr Mingozzi F. Bennicelli J. (2008). Safety and efficacy of gene transfer for Leber’s congenital amaurosis. N. Engl. J. Med. 358 (21), 2240–2248. 10.1056/NEJMOA0802315 18441370 PMC2829748

[B104] Mahato B. Kaya K. D. Fan Y. Sumien N. Shetty R. A. Zhang W. (2020). Pharmacologic fibroblast reprogramming into photoreceptors restores vision. Nature 581 (7806), 83–88. 10.1038/S41586-020-2201-4 32376950 PMC7469946

[B105] Mandai M. Watanabe A. Kurimoto Y. Hirami Y. Morinaga C. Daimon T. (2017). Autologous induced stem-cell-derived retinal cells for macular degeneration. N. Engl. J. Med. 376 (11), 1038–1046. 10.1056/NEJMOA1608368 28296613

[B106] Matsuda T. Cepko C. L. (2004). Electroporation and RNA interference in the rodent retina *in vivo* and *in vitro* . Proc. Natl. Acad. Sci. U. S. A. 101 (1), 16–22. 10.1073/PNAS.2235688100 14603031 PMC314130

[B107] McLaughlin M. E. Sandberg M. A. Berson E. L. Dryja T. P. (1993). Recessive mutations in the gene encoding the beta-subunit of rod phosphodiesterase in patients with retinitis pigmentosa. Nat. Genet. 4 (2), 130–134. 10.1038/NG0693-130 8394174

[B108] Mellough C. B. Sernagor E. Moreno-Gimeno I. Steel D. H. W. Lako M. (2012). Efficient stage-specific differentiation of human pluripotent stem cells toward retinal photoreceptor cells. Stem cells 30 (4), 673–686. 10.1002/STEM.1037 22267304

[B109] Miyagishima K. J. Wan Q. Corneo B. Sharma R. Lotfi M. R. Boles N. C. (2016). In pursuit of authenticity, induced pluripotent stem cell-derived retinal pigment epithelium for clinical applications. Stem Cells Transl. Med. 5 (11), 1562–1574. 10.5966/sctm.2016-0037 27400791 PMC5070511

[B110] Mohand-Said S. Deudon-Combe A. Hicks D. Simonutti M. Forster V. Fintz A. C. (1998). Normal retina releases a diffusible factor stimulating cone survival in the retinal degeneration mouse. Proc. Natl. Acad. Sci. U. S. A. 95 (14), 8357–8362. 10.1073/PNAS.95.14.8357 9653191 PMC20980

[B111] Mohanty S. (2012). Non-viral delivery and optimized optogenetic stimulation of retinal ganglion cells led to behavioral restoration of vision. Nat. Preced. 2012, 1. 10.1038/npre.2012.6869.1

[B112] Nau A. Bach M. Fisher C. (2013). Clinical tests of ultra-low vision used to evaluate rudimentary visual perceptions enabled by the BrainPort vision device. Transl. Vis. Sci. Technol. 2 (3), 1. 10.1167/TVST.2.3.1 PMC3763892 24049716

[B113] Nawy S. (1999). The metabotropic receptor mGluR6 may signal through G(o), but not phosphodiesterase, in retinal bipolar cells. J. Neurosci. , official J. Soc. Neurosci. 19 (8), 2938–2944. 10.1523/JNEUROSCI.19-08-02938.1999 PMC6782267 10191311

[B114] Niketeghad S. Pouratian N. (2019). Brain machine interfaces for vision restoration, the current state of cortical visual prosthetics. Neurother. , J. Am. Soc. Exp. Neurother. 16 (1), 134–143. 10.1007/S13311-018-0660-1 PMC6361050 30194614

[B115] Nuhu F. Gordon A. Sturmey R. Seymour A. M. Bhandari S. (2020). Measurement of glutathione as a tool for oxidative stress studies by high performance liquid chromatography. Molecules 25 (18), 4196. 10.3390/MOLECULES25184196 32933160 PMC7571047

[B116] O’Koren E. G. Yu C. Klingeborn M. Wong A. Y. W. Prigge C. L. Mathew R. (2019). Microglial function is distinct in different anatomical locations during retinal homeostasis and degeneration. Immunity 50 (3), 723–737. 10.1016/J.IMMUNI.2019.02.007 30850344 PMC6592635

[B117] Ostad-Ahmadi Z. Daemi A. Modabberi M. R. Mostafaie A. (2021). Safety, effectiveness, and cost-effectiveness of Argus II in patients with retinitis pigmentosa, a systematic review. Int. J. Ophthalmol. 14 (2), 310–316. 10.18240/IJO.2021.02.20 33614463 PMC7840367

[B118] Özmert E. Arslan U. (2020). Management of retinitis pigmentosa by Wharton’s jelly derived mesenchymal stem cells, preliminary clinical results. Stem Cell Res. Ther. 11 (1), 25. 10.1186/S13287-020-1549-6 31931872 PMC6958670

[B119] Palanker D. Le Mer Y. Mohand-Said S. Muqit M. Sahel J. A. (2020). Photovoltaic restoration of central vision in atrophic age-related macular degeneration. Ophthalmology 127 (8), 1097–1104. 10.1016/j.ophtha.2020.02.024 32249038 PMC7384969

[B120] Palanker D. Le Mer Y. Mohand-Said S. Sahel J. A. (2022). Simultaneous perception of prosthetic and natural vision in AMD patients. Nat. Commun. 13 (1), 513. 10.1038/s41467-022-28125-x 35082313 PMC8792035

[B121] Peng B. Xiao J. Wang K. So K. F. Tipoe G. L. Lin B. (2014). Suppression of microglial activation is neuroprotective in a mouse model of human retinitis pigmentosa. J. Neurosci. , official J. Soc. Neurosci. 34 (24), 8139–8150. 10.1523/JNEUROSCI.5200-13.2014 PMC6608244 24920619

[B122] Picaud S. Dalkara D. Marazova K. Goureau O. Roska B. Sahel J. A. (2019). The primate model for understanding and restoring vision. Proc. Natl. Acad. Sci. U. S. A. 116 (52), 26280–26287. 10.1073/PNAS.1902292116 31871177 PMC6936588

[B123] Pinilla I. Maneu V. Campello L. Fernández-Sánchez L. Martínez-Gil N. Kutsyr O. (2022). Inherited retinal dystrophies, role of oxidative stress and inflammation in their physiopathology and therapeutic implications. Antioxidants 11 (6), 1086. 10.3390/antiox11061086 35739983 PMC9219848

[B124] Pio-Lopez L. Poulkouras R. Depannemaecker D. (2021). Visual cortical prosthesis, an electrical perspective. J. Med. Eng. Technol. 45 (5), 394–407. 10.1080/03091902.2021.1907468 33843427

[B125] Pr T. (2017). “The intracortical visual prosthesis project,” in Artificial vision (Germany: Springer), 203–214.

[B126] Punzo C. Xiong W. Cepko C. L. (2012). Loss of daylight vision in retinal degeneration, are oxidative stress and metabolic dysregulation to blame? J. Biol. Chem. 287 (3), 1642–1648. 10.1074/JBC.R111.304428 22074929 PMC3265845

[B127] Qi X. Sun L. Lewin A. S. Hauswirth W. W. Guy J. (2007). Long-term suppression of neurodegeneration in chronic experimental optic neuritis, antioxidant gene therapy. Investigative Ophthalmol. Vis. Sci. 48 (12), 5360–5370. 10.1167/IOVS.07-0254 18055782

[B128] Raghu G. Berk M. Campochiaro P. A. Jaeschke H. Marenzi G. Richeldi L. (2021). The multifaceted therapeutic role of N-acetylcysteine (NAC) in disorders characterized by oxidative stress. Curr. Neuropharmacol. 19 (8), 1202–1224. 10.2174/1570159X19666201230144109 33380301 PMC8719286

[B129] Ramachandran P. S. Lee V. Wei Z. Song J. Y. Casal G. Cronin T. (2017). Evaluation of dose and safety of AAV7m8 and AAV8BP2 in the non-human primate retina. Hum. Gene Ther. 28 (2), 154–167. 10.1089/HUM.2016.111 27750461 PMC5312498

[B130] Ribeiro J. Procyk C. A. West E. L. O'Hara-Wright M. Martins M. F. Khorasani M. M. (2021). Restoration of visual function in advanced disease after transplantation of purified human pluripotent stem cell-derived cone photoreceptors. Cell Rep. 35 (3), 109022. 10.1016/J.CELREP.2021.109022 33882303 PMC8065177

[B131] Rodieck R. W. (1998). The first steps in seeing. 1st edn. Oxford: Sinauer Associates.

[B132] Roman A. J. Powers C. A. Semenov E. P. Sheplock R. Aksianiuk V. Russell R. C. (2019). Short-wavelength sensitive cone (S-cone) testing as an outcome measure for NR2E3 clinical treatment trials. Int. J. Mol. Sci. 20 (10), 2497. 10.3390/IJMS20102497 31117170 PMC6566804

[B133] Rosenfeld P. J. Cowley G. S. McGee T. L. Sandberg M. A. Berson E. L. Dryja T. P. (1992). A null mutation in the rhodopsin gene causes rod photoreceptor dysfunction and autosomal recessive retinitis pigmentosa. Nat. Genet. 1 (3), 209–213. 10.1038/NG0692-209 1303237

[B134] Rush A. D. Troyk P. R. (2012). A power and data link for a wireless-implanted neural recording system. IEEE Trans. bio-medical Eng. 59 (11), 3255–3262. 10.1109/TBME.2012.2214385 22922687

[B135] Russell S. Bennett J. Wellman J. A. Chung D. C. Yu Z. F. Tillman A. (2017). Efficacy and safety of voretigene neparvovec (AAV2-hRPE65v2) in patients with RPE65-mediated inherited retinal dystrophy, a randomised, controlled, open-label, phase 3 trial. Lancet 390, 849–860. 10.1016/S0140-6736(17)31868-8 28712537 PMC5726391

[B136] Sahel J.-A. A. Boulanger-Scemama E. Pagot C. Arleo A. Galluppi F. Martel J. N. (2021). Partial recovery of visual function in a blind patient after optogenetic therapy. Nat. Med. 27, 1223–1229. 10.1038/s41591-021-01351-4 34031601

[B137] Sahel J. A. Mohand-Said S. Léveillard T. Hicks D. Picaud S. Dreyfus H. (2001). Rod-cone interdependence, implications for therapy of photoreceptor cell diseases. Prog. Brain Res. 131, 649–661. 10.1016/S0079-6123(01)31051-8 11420978

[B138] Sakai D. Tomita H. Maeda A. (2022). Optogenetic therapy for visual restoration. Int. J. Mol. Sci. 23 (23), 15041. 10.3390/IJMS232315041 36499371 PMC9735806

[B139] Saliba R. S. Munro P. M. G. Luthert P. J. Cheetham M. E. (2002). The cellular fate of mutant rhodopsin, quality control, degradation and aggresome formation. J. Cell Sci. 115, 2907–2918. 10.1242/JCS.115.14.2907 12082151

[B140] Schimel A. M. Abraham L. Cox D. Sene A. Kraus C. Dace D. S. (2011). N-acetylcysteine amide (NACA) prevents retinal degeneration by up-regulating reduced glutathione production and reversing lipid peroxidation. Am. J. Pathology 178 (5), 2032–2043. 10.1016/J.AJPATH.2011.01.036 PMC3081196 21457933

[B141] Sharma A. Jaganathan B. G. (2021). Stem cell therapy for retinal degeneration, the evidence to date. Biol. , targets & Ther. 15, 299–306. 10.2147/BTT.S290331 PMC8327474 34349498

[B142] Shen J. Yang X. Dong A. Petters R. M. Peng Y. W. Wong F. (2005). Oxidative damage is a potential cause of cone cell death in retinitis pigmentosa. J. Cell. physiology 203 (3), 457–464. 10.1002/JCP.20346 15744744

[B143] Shin J. H. Kim H. W. Rhyu I. J. Kee S. H. (2016). Axin is expressed in mitochondria and suppresses mitochondrial ATP synthesis in HeLa cells. Exp. Cell Res. 340 (1), 12–21. 10.1016/J.YEXCR.2015.12.003 26704260

[B144] Shivdasani M. N. Sinclair N. C. Gillespie L. N. Petoe M. A. Titchener S. A. Fallon J. B. (2017). Identification of characters and localization of images using direct multiple-electrode stimulation with a suprachoroidal retinal prosthesis. Investigative Ophthalmol. Vis. Sci. 58 (10), 3962–3974. 10.1167/IOVS.16-21311 28793152

[B145] Silson E. H. Gouws A. D. Legge G. E. Morland A. B. (2022). In a case of longstanding low vision regions of visual cortex that respond to tactile stimulation of the finger with Braille characters are not causally involved in the discrimination of those same Braille characters. Cortex; a J. devoted study Nerv. Syst. Behav. 155, 277–286. 10.1016/J.CORTEX.2022.07.012 36054997

[B146] Slater K. D. Sinclair N. C. Nelson T. S. Blamey P. J. McDermott H. J. Bionic Vision Australia Consortium (2015). neuroBi, A highly configurable neurostimulator for a retinal prosthesis and other applications. IEEE J. Transl. Eng. health Med. 3, 3800111. 10.1109/JTEHM.2015.2455507 27170910 PMC4848081

[B147] Smith J. A. Das A. Ray S. K. Banik N. L. (2012). Role of pro-inflammatory cytokines released from microglia in neurodegenerative diseases. Brain Res. Bull. 87 (1), 10–20. 10.1016/J.BRAINRESBULL.2011.10.004 22024597 PMC9827422

[B148] Snodderly D. M. Weinhaus R. S. Choi J. C. (1992). Neural-vascular relationships in central retina of macaque monkeys (*Macaca fascicularis*). J. Neurosci. , official J. Soc. Neurosci. 12 (4), 1169–1193. 10.1523/JNEUROSCI.12-04-01169.1992 PMC6575794 1556592

[B149] St-Pierre J. Drori S. Uldry M. Silvaggi J. M. Rhee J. Jäger S. (2006). Suppression of reactive oxygen species and neurodegeneration by the PGC-1 transcriptional coactivators. Cell 127 (2), 397–408. 10.1016/J.CELL.2006.09.024 17055439

[B150] Stronks H. C. Mitchell E. B. Nau A. C. Barnes N. (2016). Visual task performance in the blind with the BrainPort V100 vision aid. Expert Rev. Med. devices 13 (10), 919–931. 10.1080/17434440.2016.1237287 27633972

[B151] Subhramanyam C. S. Wang C. Hu Q. Dheen S. T. (2019). Microglia-mediated neuroinflammation in neurodegenerative diseases. Seminars Cell & Dev. Biol. 94, 112–120. 10.1016/J.SEMCDB.2019.05.004 31077796

[B152] Takagi S. Mandai M. Gocho K. Hirami Y. Yamamoto M. Fujihara M. (2019). Evaluation of transplanted autologous induced pluripotent stem cell-derived retinal pigment epithelium in exudative age-related macular degeneration. Ophthalmol. Retina 3 (10), 850–859. 10.1016/J.ORET.2019.04.021 31248784

[B153] Taylor A. W. (2016). Ocular immune privilege and transplantation. Front. Immunol. 7, 37. 10.3389/FIMMU.2016.00037 26904026 PMC4744940

[B154] Toomey C. B. (2015) ‘Regulation of age-related macular degeneration-like pathology by complement factor H’, 10.1073/pnas.1424391112 PMC4466717 25991857

[B155] Tuekprakhon A. Sangkitporn S. Trinavarat A. Pawestri A. R. Vamvanij V. Ruangchainikom M. (2021). Intravitreal autologous mesenchymal stem cell transplantation, a non-randomized phase I clinical trial in patients with retinitis pigmentosa. Stem Cell Res. Ther. 12 (1), 52. 10.1186/S13287-020-02122-7 33422139 PMC7796606

[B156] Tzekov R. Stein L. Kausha S. (2011). Protein misfolding and retinal degeneration. Cold Spring Harb. Perspect. Biol. 3 (11), a007492. 10.1101/CSHPERSPECT.A007492 21825021 PMC3220361

[B157] Usui S. Komeima K. Lee S. Y. Jo Y. J. Ueno S. Rogers B. S. (2009). Increased expression of catalase and superoxide dismutase 2 reduces cone cell death in retinitis pigmentosa. Mol. Ther. , J. Am. Soc. Gene Ther. 17 (5), 778–786. 10.1038/MT.2009.47 PMC2803613 19293779

[B158] Vaidya A. Borgonovi E. Taylor R. S. Sahel J. A. Rizzo S. Stanga P. E. (2014). The cost-effectiveness of the Argus II retinal prosthesis in Retinitis Pigmentosa patients. BMC Ophthalmol. 14 (1), 49. 10.1186/1471-2415-14-49 24731533 PMC3990272

[B159] Vandenberghe L. H. Bell P. Maguire A. M. Cearley C. N. Xiao R. Calcedo R. (2011). Dosage thresholds for AAV2 and AAV8 photoreceptor gene therapy in monkey. Sci. Transl. Med. 3, 88ra54. 10.1126/SCITRANSLMED.3002103 PMC5027886 21697530

[B160] Veske A. Nilsson S. E. Narfström K. Gal A. (1999). Retinal dystrophy of Swedish briard/briard-beagle dogs is due to a 4-bp deletion in RPE65. Genomics 57 (1), 57–61. 10.1006/GENO.1999.5754 10191083

[B161] Vighi E. Trifunović D. Veiga-Crespo P. Rentsch A. Hoffmann D. Sahaboglu A. (2018). Combination of cGMP analogue and drug delivery system provides functional protection in hereditary retinal degeneration. Proc. Natl. Acad. Sci. U. S. A. 115 (13), E2997–E3006. 10.1073/PNAS.1718792115 29531030 PMC5879685

[B162] Wang S. K. Xue Y. Cepko C. L. (2021). Augmentation of CD47/SIRPα signaling protects cones in genetic models of retinal degeneration. JCI insight 6 (16), e150796. 10.1172/JCI.INSIGHT.150796 34197341 PMC8409989

[B163] Wang S. K. Xue Y. Cepko C. L. (2020). Microglia modulation by TGF-β1 protects cones in mouse models of retinal degeneration. J. Clin. investigation 130 (8), 4360–4369. 10.1172/JCI136160 PMC7410072 32352930

[B164] Wang X. Iannaccone A. Jablonski M. M. (2003). Permissive glycan support of photoreceptor outer segment assembly occurs via a non-metabolic mechanism. Mol. Vis. 9, 701–709. 14685143

[B165] Wang Y. (2022). Tsc2 knockout counteracts ubiquitin-proteasome system insufficiency and delays photoreceptor loss in retinitis pigmentosa. Proc. Natl. Acad. Sci. U. S. A. 119 (11). 10.1073/PNAS.2118479119 PMC8931319 35275792

[B166] Watari K. Yamasaki S. Tu H. Y. Shikamura M. Kamei T. Adachi H. (2023). Self-organization, quality control, and preclinical studies of human iPSC-derived retinal sheets for tissue-transplantation therapy. Commun. Biol. 6 (1), 164. 10.1038/S42003-023-04543-5 36765170 PMC9918541

[B167] Wright A. F. (1997). A searchlight through the fog. Nat. Genet. 17 (2), 132–134. 10.1038/NG1097-132 9326923

[B168] Wright W. W. Gajjeraman S. Batabyal S. Pradhan S. Bhattacharya S. Mahapatra V. (2017). Restoring vision in mice with retinal degeneration usingmulticharacteristic opsin. Neurophotonics 4 (4), 041505. 10.1117/1.NPH.4.4.041505 28948190 PMC5603575

[B169] Wu D. M. Ji X. Ivanchenko M. V. Chung M. Piper M. Rana P. (2021). Nrf2 overexpression rescues the RPE in mouse models of retinitis pigmentosa. JCI insight 6 (2), e145029. 10.1172/JCI.INSIGHT.145029 33491671 PMC7934854

[B170] Xiong W. Wu D. M. Xue Y. Wang S. K. Chung M. J. Ji X. (2019). AAV cis-regulatory sequences are correlated with ocular toxicity. Proc. Natl. Acad. Sci. U. S. A. 116 (12), 5785–5794. 10.1073/PNAS.1821000116 30833387 PMC6431174

[B171] Yamasaki R. Lu H. Butovsky O. Ohno N. Rietsch A. M. Cialic R. (2014). Differential roles of microglia and monocytes in the inflamed central nervous system. J. Exp. Med. 211 (8), 1533–1549. 10.1084/JEM.20132477 25002752 PMC4113947

[B172] Yang Y. Mohand-Said S. Danan A. Simonutti M. Fontaine V. Clerin E. (2009). Functional cone rescue by RdCVF protein in a dominant model of retinitis pigmentosa. Mol. Ther. , J. Am. Soc. Gene Ther. 17 (5), 787–795. 10.1038/MT.2009.28 PMC2835133 19277021

[B173] Yu D. Y. Cringle S. Valter K. Walsh N. Lee D. Stone J. (2004). Photoreceptor death, trophic factor expression, retinal oxygen status, and photoreceptor function in the P23H rat. Investigative Ophthalmol. Vis. Sci. 45 (6), 2013–2019. 10.1167/IOVS.03-0845 15161870

[B174] Zaghloul N. A. Katsanis N. (2009). Mechanistic insights into Bardet-Biedl syndrome, a model ciliopathy. J. Clin. investigation 119 (3), 428–437. 10.1172/JCI37041 PMC2648685 19252258

[B175] Zerti D. Hilgen G. Dorgau B. Collin J. Ader M. Armstrong L. (2021). Transplanted pluripotent stem cell-derived photoreceptor precursors elicit conventional and unusual light responses in mice with advanced retinal degeneration. Stem cells 39 (7), 882–896. 10.1002/STEM.3365 33657251

[B176] Zhao L. Zabel M. K. Wang X. Ma W. Shah P. Fariss R. N. (2015). Microglial phagocytosis of living photoreceptors contributes to inherited retinal degeneration. EMBO Mol. Med. 7 (9), 1179–1197. 10.15252/EMMM.201505298 26139610 PMC4568951

[B177] Zhao T. Liang Q. Meng X. Duan P. Wang F. Li S. (2020). Intravenous infusion of umbilical cord mesenchymal stem cells maintains and partially improves visual function in patients with advanced retinitis pigmentosa. Stem cells Dev. 29 (16), 1029–1037. 10.1089/SCD.2020.0037 32679004

[B178] Zhong X. Gutierrez C. Xue T. Hampton C. Vergara M. N. Cao L. H. (2014). Generation of three-dimensional retinal tissue with functional photoreceptors from human iPSCs. Nat. Commun. 5, 4047. 10.1038/NCOMMS5047 24915161 PMC4370190

[B179] Zhou D. D. Dorn J. D. Greenberg R. J. (2013). “The Argus® II retinal prosthesis system, an overview,” in Electronic Proceedings of the 2013 IEEE International Conference on Multimedia and Expo Workshops, 15-19 July 2013. *ICMEW 2013* [Preprint]. 10.1109/ICMEW.2013.6618428

[B180] Zhu X. Ma B. Babu S. Murage J. Knox B. E. Craft C. M. (2002). Mouse cone arrestin gene characterization, Promoter targets expression to cone photoreceptors. FEBS Lett. 524 (1–3), 116–122. 10.1016/S0014-5793(02)03014-4 12135752

